# Genetic Association and Causal Effects Between Obesity and Multiple Sclerosis: A Robust Two-Sample Mendelian Randomization Study

**DOI:** 10.7759/cureus.89287

**Published:** 2025-08-03

**Authors:** Abdulaziz Almosallam

**Affiliations:** 1 Internal Medicine, Majmaah University, Al Majma'ah, SAU

**Keywords:** body mass index, causal effects, genetic association, mendelian randomization, multiple sclerosis

## Abstract

Background: A relationship between obesity, as measured by body mass index (BMI), and multiple sclerosis (MS) has been reported in several observational studies. This study aimed to investigate the potential causal relationship between BMI and the risk of developing MS using a Mendelian randomization (MR) approach.

Materials and methods: A two-sample MR analysis was performed using single nucleotide polymorphisms (SNPs) associated with the exposures - BMI and MS - sourced from publicly available genome-wide association studies (GWAS) at a genome-wide significance threshold of *P* = 5 × 10^-8^. The primary analytical methods included inverse-variance weighted (IVW), weighted median, MR-Egger, simple mode, and weighted mode approaches. Cochran's Q-test was applied to assess heterogeneity, while the MR-Egger intercept was used to detect potential horizontal pleiotropy. Sensitivity and robustness were evaluated through leave-one-out analysis. Additional methods - radial IVW, radial MR-Egger, MR-Pleiotropy RESidual Sum and Outlier (MR-PRESSO), MR-Least Absolute Shrinkage and Selection Operator (MR-LASSO), Generalized Summary-data-based Mendelian Randomization v2 (GSMR2), and MR-Robust Adjusted Profile Score (MR-RAPS) - were employed to estimate causal effects, assess instrument validity, and identify potential outlier SNPs. A *P*-value of less than 0.05 was considered statistically significant.

Results: The MR analysis revealed a causal effect of BMI on MS as indicated by IVW (odds ratio (OR) = 1.39; 95% confidence interval (CI) = 1.23, 1.58; P = 1.42e-07), IVW method with modified second-order weights (IVW (Mod.2nd)) (OR = 1.39; 95% CI = 1.28, 1.51; P = 2.31e-15), MR-Egger (OR = 1.39; 95% CI = 1.01, 1.92; P = 4.51e-02), MR-Genotype Recoding Invariance Property (MR-GRIP) (OR = 1.41; 95% CI = 1.21, 1.65; P = 1.80e-05), MR-PRESSO (OR = 1.39; 95% CI = 1.28, 1.51; P = 3.79e-14), MR-RAPS (OR = 1.39; 95% CI = 1.24, 1.56; P = 2.66e-08), and GSMR (OR = 1.38; 95% CI = 1.23, 1.55; P = 3.35e-08). No evidence of horizontal pleiotropy or heterogeneity was detected, and the findings were confirmed to be robust.

Conclusion: This study provides evidence that an increase in body size (BMI) can lead to MS. Therefore, maintaining a healthy weight during early life could be a potential strategy to reduce MS risk.

## Introduction

Multiple sclerosis (MS) is a chronic inflammatory disorder of the central nervous system (CNS) that primarily affects the white matter of the brain, spinal cord, and optic nerves. It has both genetic and environmental factors contributing to its development [1]. Observational studies, while valuable for identifying potential risk factors, are often insufficient on their own for definitively clarifying causal relationships in MS due to limitations like confounding, reverse causality, and lack of control over exposures. MS is characterized by a female predominance and an estimated prevalence of 30-190 cases per 100,000 people. The incidence rate per 100,000 in Europe is documented [2]. Worldwide, nearly 2.8 million people are affected by MS, with an incidence rate of 35.9 per 100,000 individuals [3]. Higher levels of serum 25-hydroxyvitamin D appear to reduce the risk of MS [4,5]. Recently, the link between MS and obesity has gained increased attention [6]. Emerging research suggests a potential connection between body mass index (BMI) and both the risk of developing MS and disease progression, with obesity doubling the risk of MS in late adolescence or early adulthood (around age 18) [7]. Patients with higher BMI tend to experience more frequent and severe relapses [8]. Current evidence indicates a causal relationship and shared genetic factors between BMI and MS [9]. This association may be due to various mechanisms, including chronic low-grade inflammation common in obesity, which could potentially trigger autoimmunity [10].

Furthermore, excess body weight can affect the progression of MS after diagnosis. Studies show that overweight and obese individuals with MS may experience more severe disease progression, higher relapse rates, and greater disability compared to those with normal weight [6,11-13]. This highlights the importance of weight management as a vital component of comprehensive MS treatment. Clinically, these findings underscore the crucial role of healthcare providers in assessing and advising patients on maintaining a healthy weight. Including BMI assessments in routine evaluations could help reduce relapse rates and improve long-term outcomes. Because multiple causes contribute to MS, a comprehensive treatment approach that incorporates lifestyle changes is essential [14,15]. The link between BMI and MS provides valuable insights for clinicians managing this complex disease. Increased awareness of this connection not only facilitates preventive efforts but also improves the overall management of MS.

Mendelian randomization (MR) studies have garnered significant attention due to their ability to provide detailed insights into causal relationships, particularly when randomized controlled trial designs are not feasible [16-18]. They offer a strong and dependable method for better understanding the causality between exposure (risk factor) and outcome (disease). The current study aimed to examine the genetic correlation, causal connections, and shared risk loci with potential functions between obesity and MS, to clarify their comorbidity.

## Materials and methods

Data sources

**Figure 1:**
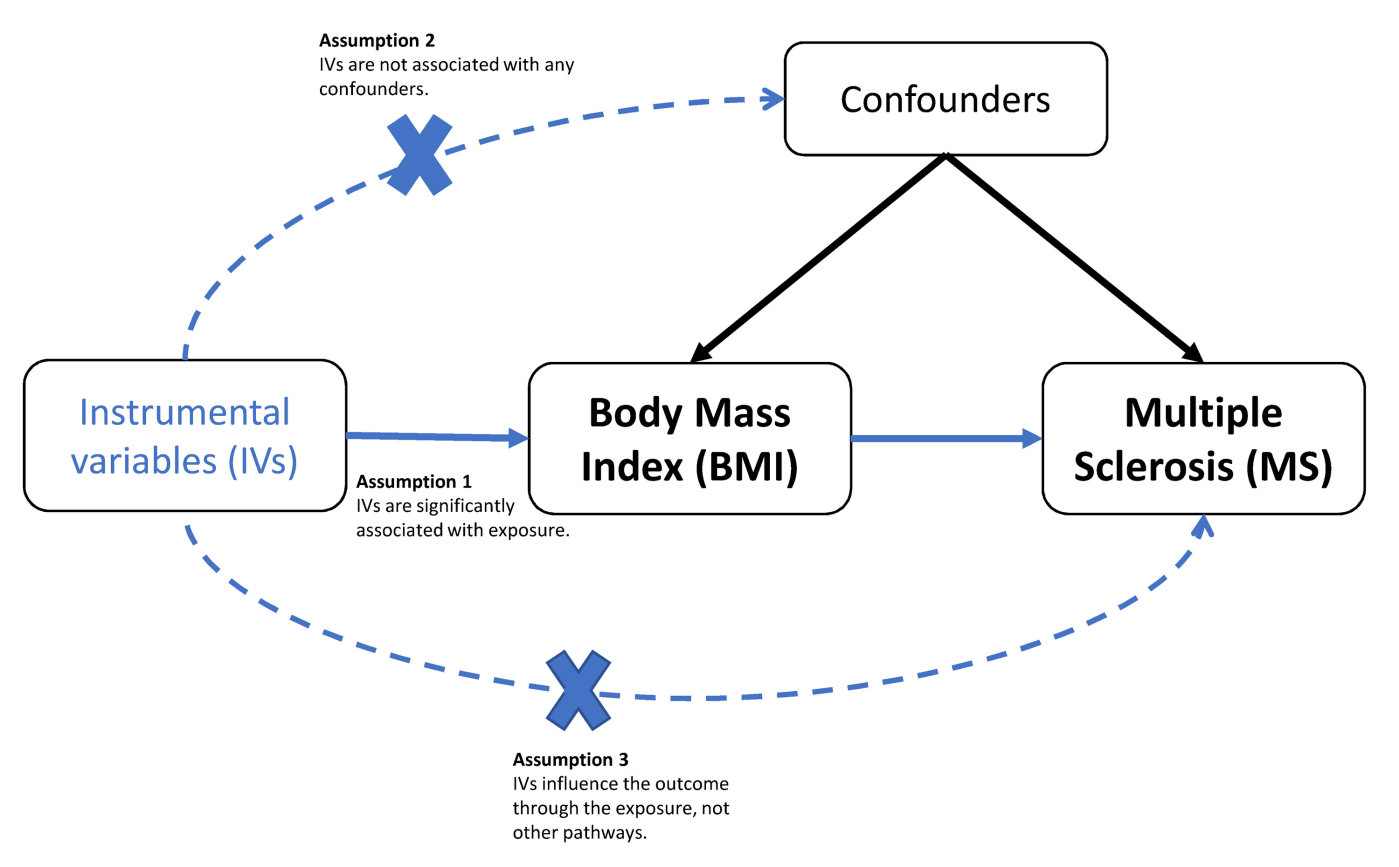
Schematic representation of the study design for the current MR analysis, highlighting the three core assumptions. MR: Mendelian randomization Image credits: Author Abdulaziz Almosallam

This study used publicly available genome-wide association study (GWAS) summary statistics obtained from ethically approved research where informed consent was obtained from all original participants. The analysis was conducted in accordance with the Strengthening the Reporting of Observational Studies in Epidemiology-Mendelian Randomization (STROBE-MR) guidelines [19]. As the study utilized only summary-level data, no further ethical approval was required. The study protocol was not formally registered. The key assumptions underlying the MR design are illustrated in Figure 1.

Data were sourced from the IEU OpenGWAS database (https://gwas.mrcieu.ac.uk/), accessed on June 21, 2025, including the following datasets. (i) ukb-b-19953 (BMI): This dataset contains 461,460 individuals of European ancestry, totaling 9,851,867 single nucleotide polymorphisms (SNPs). (ii) ieu-b-18 (MS): This dataset includes a total sample size of 115,803 individuals from the European population, with 6,304,359 SNPs. Both datasets include participants of both sexes, providing a comprehensive basis for the two-sample MR analysis. More details are shown in Table 1.

**Table 1:** Characteristics of the traits used in the study. SNPs: Single nucleotide polymorphisms; MRC-IEU: Medical Research Council Integrative Epidemiology Unit; IVs: Instrumental variables; GWAS: Genome-wide association study

Trait's name	Body mass index (BMI)	Multiple sclerosis (MS)
GWAS id	ukb-b-19953	ieu-b-18
Population	European	European
Year	2018	2019
Sample size	461,460	115,803
Sex	Male and female	Male and female
No. of SNPs	9,851,867	6,304,359
Author	Ben Elsworth	Nikolaos A. Patsopoulos
Consortium	MRC-IEU	International Multiple Sclerosis Genetics Consortium
Retrieved SNPs	458 SNPs (19 were removed during clumping)	453 SNPs
Harmonized SNPs	439 SNPs: 21 SNPs were removed for being palindromic, and another 95 IVs were removed for being weak. The final analysis included 323 SNPs	

Data extraction

We implemented a two-sample MR analysis. A genome-wide significance threshold of *P *= 5 × 10^-8^ was applied to identify instrumental variables (IVs) associated with the exposure. For the analysis direction, SNPs from the ukb-b-19953 dataset were used as the exposure (BMI), while SNPs from the ieu-b-18 dataset (MS) represented the outcome. A total of 458 SNPs were extracted from the exposure data, which were clumped with clump_kb = 10000 and clump_r2 = 0.001. Using the European population reference, 19 of 458 variants were removed due to linkage disequilibrium (LD) with other variants or absence from the LD reference panel. Extraction of the outcome revealed 453 SNPs. After harmonizing the data, 21 SNPs were removed for being palindromic with intermediate allele frequencies. SNPs with weak instrument strength (n = 95) were also detected using the constrained maximum likelihood (MRcML) method and subsequently removed. Finally, a total of 323 SNPs were included for the final analysis. Detailed information regarding the selected SNPs and their characteristics can be found in Supplementary File 1.

Data analysis

The primary analysis employed the radial inverse-variance weighted (IVW) method with modified second-order weights (IVW (Mod.2nd)) to identify heterogeneous genetic variants and estimate causal effects. Complementary MR methods included the weighted median, which yields reliable causal estimates even when up to 50% of the genetic instruments are invalid [20], and the weighted mode, which remains consistent even if more than 50% of the instruments are invalid [21]. To address the influence of weak instruments and outlier variants, the MR-Robust Adjusted Profile Score (MR-RAPS) was applied [22]. The simple mode method was also used to estimate causal effects based on the cluster containing the largest number of SNPs [23]. Additional methods employed for causal inference included standard IVW, MR-Egger, radial MR-Egger, MR-Genotype Recoding Invariance Property (MR-GRIP), Generalized Summary-data-based Mendelian Randomization v2 (GSMR2), and MR-Least Absolute Shrinkage and Selection Operator (MR-LASSO). To identify outlier variants that potentially bias the pooled SNP effect in IVW estimates, MR-RAPS, MR-Pleiotropy RESidual Sum and Outlier (PRESSO), and the leave-one-out approach were employed [24]. Additionally, heterogeneity among individual SNPs was assessed using Cochran's Q-test (P < 0.05 indicating heterogeneity), the MR-Egger regression intercept (P < 0.05 indicating heterogeneity), and the MR-PRESSO global test, which evaluates the observed residual sum of squares (RSSobs), with P < 0.05 similarly indicating heterogeneity.

Causal estimates were presented as odds ratios (OR) with 95% confidence intervals (CI). All statistical analyses were conducted in R version 4.3 (R Foundation for Statistical Computing, Vienna, Austria) using the "TwoSampleMR" (version 0.6.16), "MendelianRandomization" (version 4.4.3), "RadialMR" (version 1.2), "MRPRESSO" (version 4.4.3), and "mr.raps" (version 0.4.2) packages.

## Results

**Figure 2:**
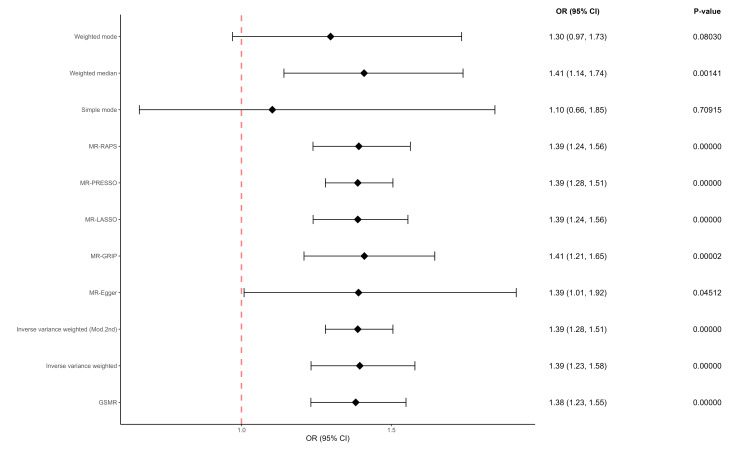
Causal estimates expressed as OR to represent the effect of BMI on the risk of MS. BMI: Body mass index; MS: Multiple sclerosis; MR: Mendelian randomization; MR-PRESSO: MR-Pleiotropy RESidual Sum and Outlier; GSMR: Generalized Summary-data-based Mendelian Randomization; MR-RAPS: MR-Robust Adjusted Profile Score; MR-LASSO: MR-Least Absolute Shrinkage and Selection Operator; MR-GRIP: MR-Genotype Recoding Invariance Property; Mod.2nd: Modified second-order weights; OR: Odds ratio; CI: Confidence interval

**Figure 3:**
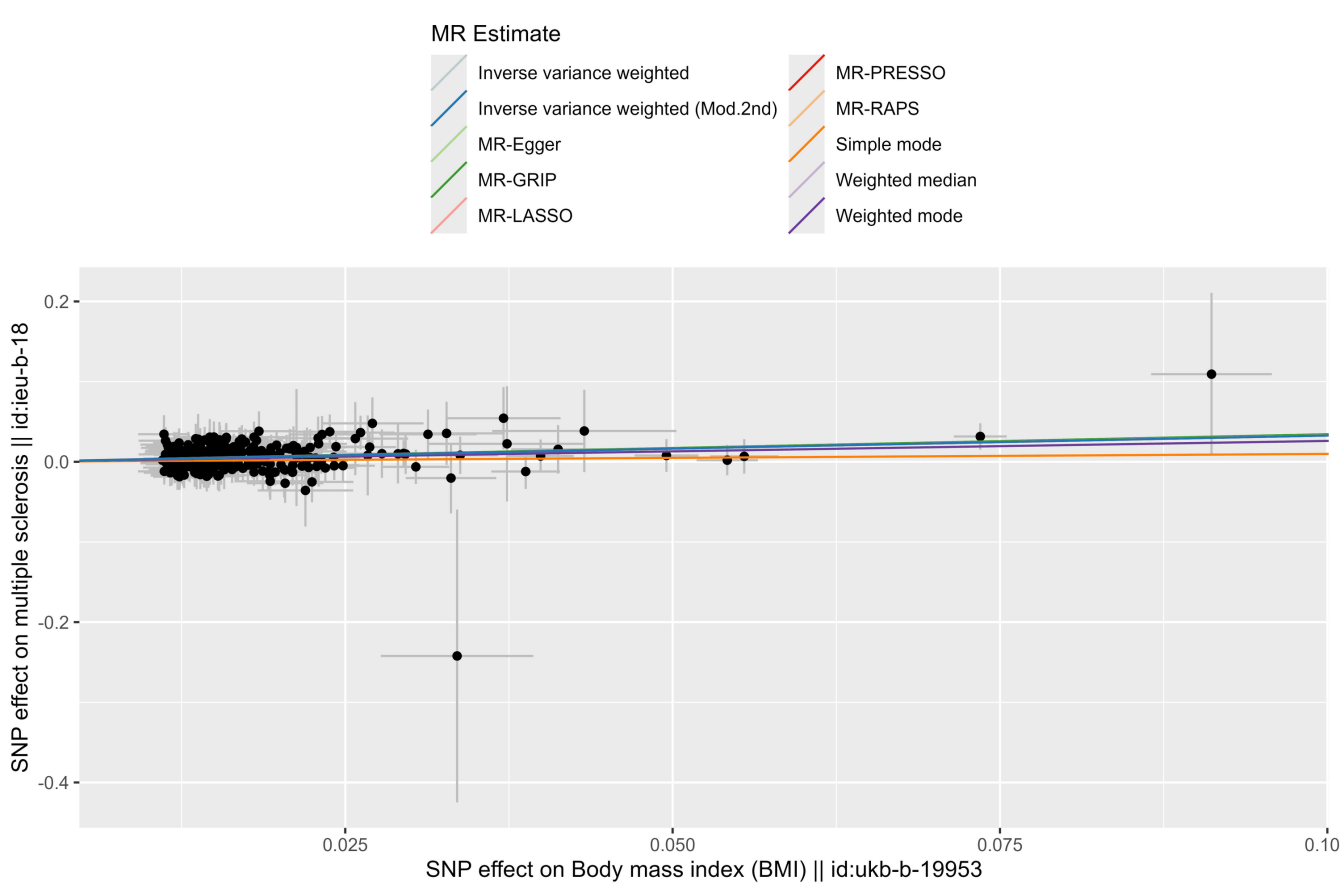
Scatter plot of genetic association between BMI and MS. A cross around each SNP shows the 95% CI. The slopes of each line represent the magnitude of the causal association for each method. BMI: Body mass index; MS: Multiple sclerosis; MR: Mendelian randomization; MR-PRESSO: MR-Pleiotropy RESidual Sum and Outlier; GSMR: Generalized Summary-data-based Mendelian Randomization; MR-RAPS: MR-Robust Adjusted Profile Score; MR-LASSO: MR-Least Absolute Shrinkage and Selection Operator; MR-GRIP: MR-Genotype Recoding Invariance Property; SNP: Single nucleotide polymorphism; Mod.2nd: Modified second-order weights

The estimated causal effects of BMI on MS are shown in Figures 2-3. There was a significant increase in MS risk as indicated by IVW (OR = 1.39; 95% CI = 1.23, 1.58; P = 1.42e-07), IVW (Mod.2nd) (OR = 1.39; 95% CI = 1.28, 1.51; P = 2.31e-15), MR-Egger (OR = 1.39; 95% CI = 1.01, 1.92; P = 4.51e-02), MR-GRIP (OR = 1.41; 95% CI = 1.21, 1.65; P = 1.80e-05), MR-LASSO (OR = 1.39; 95% CI = 1.24, 1.56; P < 0.00001), MR-PRESSO (OR = 1.39; 95% CI = 1.28, 1.51; P = 3.79e-14), MR-RAPS (OR = 1.39; 95% CI = 1.24, 1.56; P = 2.66e-08), GSMR (OR = 1.38; 95% CI = 1.23, 1.55; P = 3.35e-08), and the weighted median method (OR = 1.41; 95% CI = 1.14, 1.74; P = 1.41e-03).

Table 2 summarizes the sensitivity analyses, which revealed no evidence of heterogeneity, horizontal pleiotropy, or outliers. Cochran's Q statistic was 2.72 (degree of freedom (df) = 7, P = 0.909) for the MR-Egger method and 3.16 (df = 8, P = 0.924) for the IVW method, indicating the absence of heterogeneity in both methods.

**Table 2:** Results of sensitivity analyses. MR: Mendelian randomization; MR-PRESSO: MR-Pleiotropy RESidual Sum and Outlier; RSSobs: Observed residual sum of squares; pval: P-value; se: Standard error; df: Degree of freedom

Sensitivity analyses			
Method	Cochran's Q	Q df	Q pval
MR-Egger	2.72339	7	0.90936
Inverse-variance weighted	3.16336	8	0.92369
Method	Egger intercept	se	pval
Pleiotropy	0.00006	0.0021	0.98398
Method	RSSobs		pval
MR-PRESSO global test	163.6182		1

Furthermore, the MR-Egger intercept was close to zero with a P-value greater than 0.05, indicating no evidence of horizontal pleiotropy (Table 3). The MR-PRESSO global test yielded an RSSobs value of 163.62 with a P-value of 1.000, further supporting the absence of pleiotropic effects (Table 2).

**Table 3:** Radial estimates of MR-Egger and IVW models. MR: Mendelian randomization; IVW: Inverse-variance weighted; Mod.2nd: Modified second-order weights; df: Degree of freedom; FE: Fixed effect; RE: Random effect

Radial estimates	Estimate	Standard error	t value	Pr(>|t|)	F-statistic	df	P-value	Q-statistic	df	P-value
MR-Egger										
(Intercept)	-0.04864	0.10755	-0.45223	0.65141	11.1	321	0.00097	162.24	321	1.000
Wj	0.37528	0.11266	3.33112	0.00097						
IVW effect										
Effect (Mod.2nd)	0.32790	0.04138	7.92363	2.31e-15	62.78	322	3.8e-14	162.42	322	1.000
Exact (FE)	0.33044	0.05827	5.67010	1.42e-08						
Exact (RE)	0.33044	0.03976	8.31077	2.66e-15						

**Figure 4:**
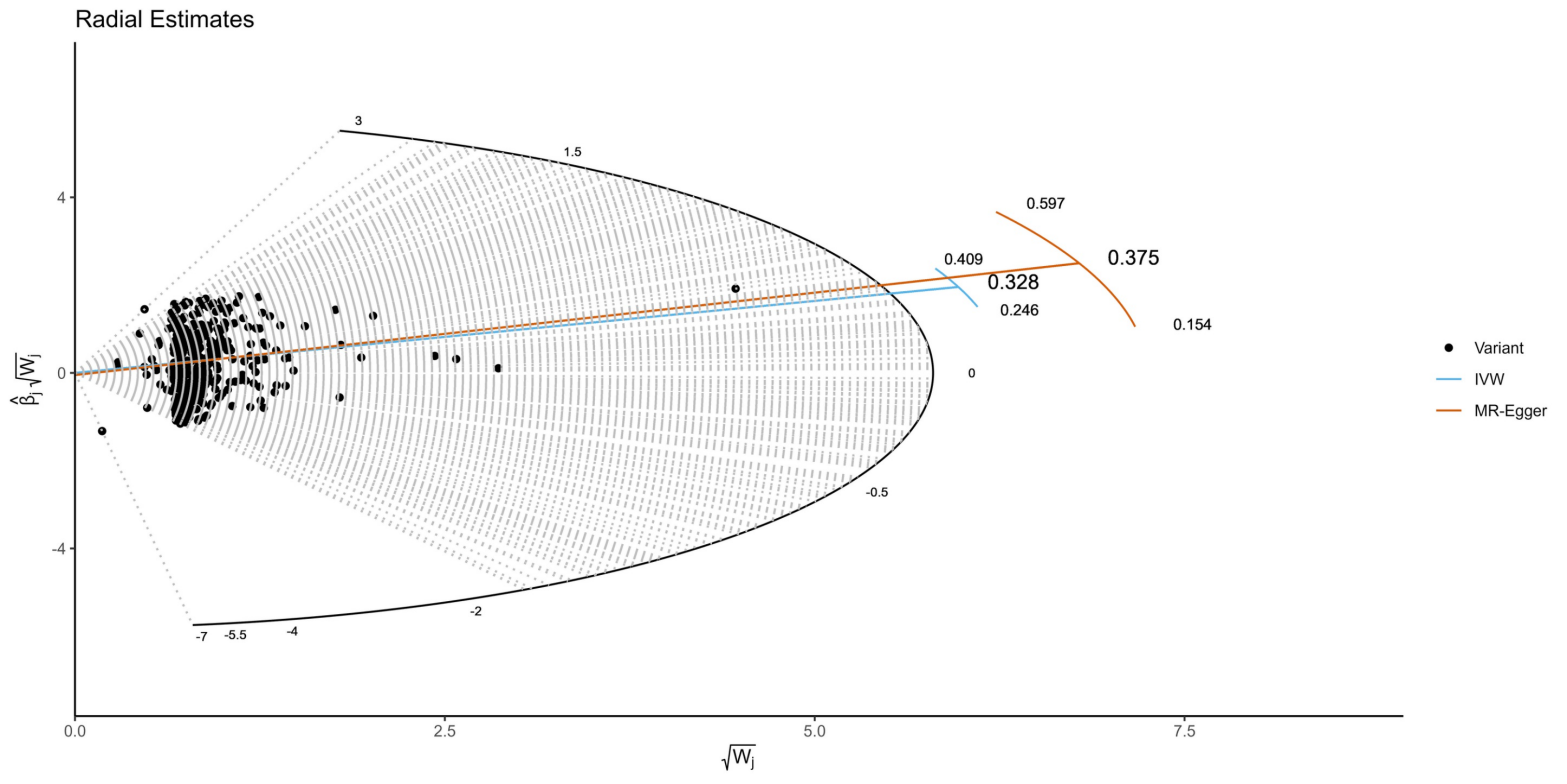
Radial plot was used to visualize individual outlier SNPs in the MR estimates assessing the effect of BMI on MS. BMI: Body mass index; MS: Multiple sclerosis; MR: Mendelian randomization; SNPs: Single nucleotide polymorphisms; IVW: Inverse-variance weighted

Furthermore, no significant outliers were identified by the radial IVW and MR-Egger models (Figure 4), and the MR-LASSO analysis confirmed that all instruments were valid. The leave-one-out test demonstrated consistency across the data (Supplementary File 2).

## Discussion

The current MR study provides strong evidence that a higher BMI is associated with an increased risk of MS. A total of 323 reliable genetic markers were identified through large-scale GWAS, demonstrating significant results across various methods, including IVW, MR-Egger, MR-RAPS, GSMR, and MR-LASSO. These findings support and reinforce previous observational studies suggesting that obesity, especially during adolescence and early adulthood, may elevate the risk of MS [6,7,14,25-30]. Most ORs of the current findings ranged from 1.38 to 1.41, indicating that higher BMI significantly increases the likelihood of MS. This association is independent of confounding factors and reverse causality, which are often difficult to rule out in traditional observational studies. MS can be associated with weight gain and obesity, but MS itself doesn't directly cause obesity; its symptoms and treatment can lead to weight fluctuations and increased risk of becoming overweight or obese. The absence of pleiotropy and heterogeneity, as indicated by Cochran's Q, MR-Egger intercept, and MR-PRESSO global test (all with nonsignificant p-values), further affirms the reliability of these results. Additionally, the consistency seen across sensitivity analyses, including leave-one-out and radial plots, supports the robustness of the association and the validity of the IVs.

From a pathophysiological perspective, these observations are biologically plausible. Obesity is known to promote systemic low-grade inflammation and alter immune function, potentially increasing the risk for autoimmune disorders such as MS [10,31]. Adipose tissue secretes pro-inflammatory cytokines (e.g., IL-6, TNF-α) and leptin, both of which can impair immune tolerance and promote autoreactive T-cell activity - a hallmark of MS pathology [31-33]. Additionally, vitamin D deficiency, often linked with obesity, may further contribute to MS risk due to its immunomodulatory functions [1,4,34]. These findings also support previous epidemiological evidence. Adiposity causes chronic low-grade inflammation. Obesity is associated with elevated levels of IL-6, TNF-α, CRP, and leptin, as well as altered macrophage profiles - all of which contribute to systemic and neural inflammation [31-33]. Obesity also correlates with decreased vitamin D bioavailability, making vitamin D deficiency an independent MS risk factor [5]. In a high-quality cohort from Sweden, BMI increase between the ages of 16 and 20 was linked to a higher risk of MS; notably, the risk continued to rise even below the thresholds that define obesity, thus demonstrating a dose-response relationship [35]. Another large Danish cohort found that for each single BMI z-score increase at ages 7-13, the risk of MS also increased [36]. It has been reported that those who were overweight at an earlier age developed MS earlier than those who were not overweight at that age [37,38]. Similarly, women reporting a larger silhouette at age 20 had an approximately 70-96% increased risk of MS [28]. A recent study showed that being overweight and obese during childhood, late childhood, or adolescence was associated with pooled relative risks for MS. This association was strong among female individuals [38]. A case-control study from Saudi Arabia found that larger adolescent silhouettes increased MS risk by over threefold [24].

These observational studies collectively show that an increase in BMI during adolescence and young adulthood is a significant risk factor for MS, especially in female individuals and with earlier age at onset. Nonetheless, these studies controlled for confounders such as smoking, sun exposure, and heredity, suggesting that other mechanisms may also be involved. Obesity triggers systemic inflammation (e.g., increased CRP, IL-6, and leptin) and reduces vitamin D availability, both of which are implicated in MS development. Although these studies involved large sample sizes, prospective designs, and adjustments for established MS risk factors, key limitations include reliance on self-reported BMI, potential residual confounding, and a predominance of female or European-ancestry populations, which limits generalizability. However, the shared observational evidence increases our confidence that elevation of BMI during childhood, adolescence, and early adulthood is linked to a significantly higher risk of developing MS, particularly among female individuals. Coupled with strong biological mechanisms (chronic inflammation, adipokine imbalance, vitamin D deficiency), this observational data reinforces the causal inference suggested by MR studies. These findings suggest that weight management in early life may serve as a preventive strategy against MS.

Clinically, these findings emphasize the importance of early intervention and weight management, especially in high-risk populations or those with a family history of MS [15,26,27,39,40]. It has been reported that a higher BMI is associated with mobility impairment, decreased self-rated health, lower health-related quality of life (HRQoL), and a more rapid decline in HRQoL [12,41]. Because there is potential for risk reduction through modifiable factors, including nutritional counseling and physical activity, incorporating these into adolescent and early adult health strategies could help prevent MS [39,40]. Given the causal relationship between BMI and MS and mechanistic insights, early weight management could serve as an effective MS prevention approach, particularly during adolescence. Clinicians should consider individualized weight management plans that focus on nutrition, physical activity, and mental well-being. Importantly, strategies should be tailored to meet each patient’s specific needs, limitations, and preferences, ensuring that lifestyle changes are practical and sustainable. Furthermore, ongoing education about the impact of BMI on MS should be promoted in both clinical settings and patient outreach programs. Engaging patients in discussions about how body weight can influence their disease can empower them to actively participate in managing their health.

Nevertheless, this study has some limitations. Although the MR design reduces confounding and reverse causality, it depends on the assumption of no horizontal pleiotropy and that the genetic instruments affect the outcome only through the exposure. While our analysis did not find strong evidence of pleiotropy, minor undetected violations might still be present. Additionally, the study was limited to individuals of European ancestry, which limits the broad applicability of the findings to other ethnic groups. Future research should aim to replicate these results in more diverse populations and further explore potential mechanistic pathways.

## Conclusions

This two-sample MR study provides strong evidence for a causal relationship between elevated BMI and increased risk of MS. These findings suggest that maintaining a healthy weight during early life could be a potential strategy to reduce MS risk. Therefore, reducing obesity might be a practical approach for MS primary prevention and overall management.

## Figures and Tables

**Figure 5:**
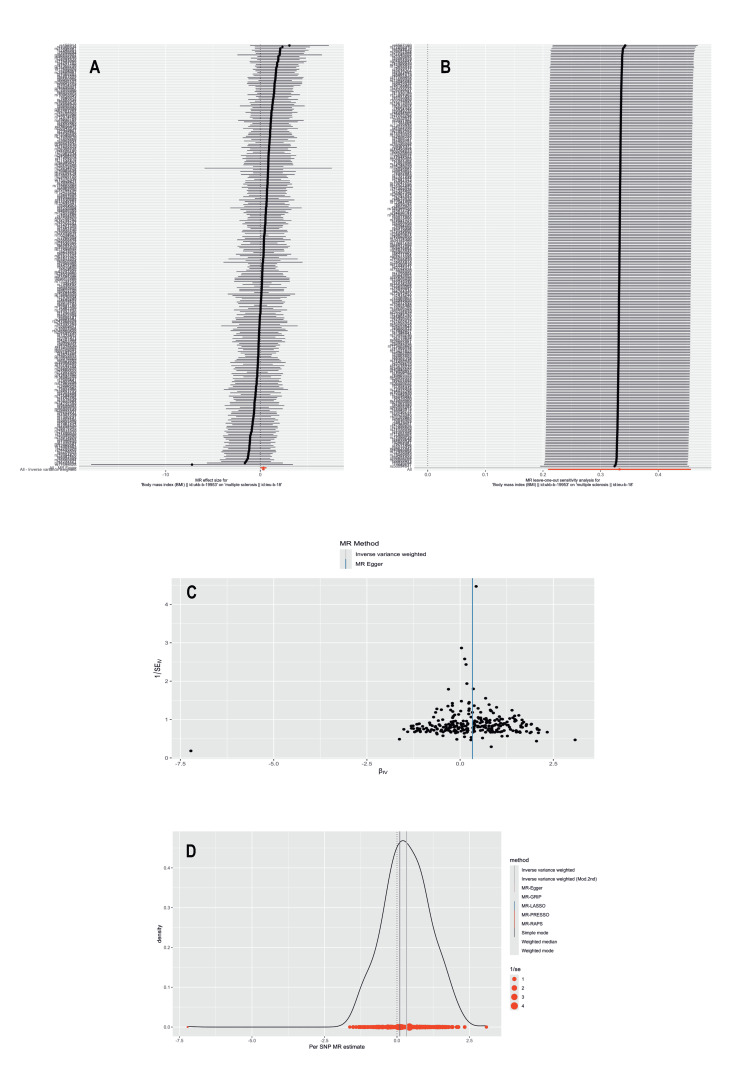
Sensitivity analyses. (A) Forest plot of single SNPs associated with BMI and their risk of MS. (B) Forest plot of leave-one-out sensitivity analysis for BMI and their risk of MS. (C) Funnel plot showing the relationship between the causal effect of BMI on MS estimated by each genetic variant against the inverse standard error of the causal estimate. (D) MR density plot to visualize the overall heterogeneity of MR estimates for the effect of BMI on MS. SNP: Single-nucleotide polymorphism; BMI: Body mass index; MS: Multiple sclerosis; MR: Mendelian randomization; MR-PRESSO: MR-Pleiotropy RESidual Sum and Outlier; MR-RAPS: MR-Robust Adjusted Profile Score; MR-LASSO: MR-Least Absolute Shrinkage and Selection Operator; MR-GRIP: MR-Genotype Recoding Invariance Property; Mod.2nd: Modified second-order weights

## Appendices

Supplementary File 1

**Table 4:** Raw values of the harmonized data for the effect of BMI on MS. SNP: Single-nucleotide polymorphism; BMI: Body mass index; MS: Multiple sclerosis

SNP	beta.exposure	beta.outcome	id.outcome	se.outcome	samplesize.outcome	pval.outcome	outcome	samplesize.exposure	se.exposure	pval.exposure	id.exposure	exposure
rs10063055	0.01367750	0.01938670	ieu-b-18	0.0192984	115803	0.3151000	Multiple Sclerosis	461460	0.00226991	1.700000E-09	ukb-b-19953	Body mass index (BMI)
rs10099330	0.01242470	0.04667240	ieu-b-18	0.0163384	115803	0.0042820	Multiple Sclerosis	461460	0.00198907	4.200010E-10	ukb-b-19953	Body mass index (BMI)
rs10160769	-0.01558620	-0.01656210	ieu-b-18	0.0193892	115803	0.3930000	Multiple Sclerosis	461460	0.00242199	1.200000E-10	ukb-b-19953	Body mass index (BMI)
rs10169594	0.01218750	-0.01714620	ieu-b-18	0.0178807	115803	0.3376000	Multiple Sclerosis	461460	0.00205506	2.999990E-09	ukb-b-19953	Body mass index (BMI)
rs10182416	0.01303780	0.01724790	ieu-b-18	0.0162431	115803	0.2883000	Multiple Sclerosis	461460	0.00197134	3.699990E-11	ukb-b-19953	Body mass index (BMI)
rs10423928	-0.03401360	-0.07194850	ieu-b-18	0.0212325	115803	0.0007025	Multiple Sclerosis	461460	0.00249869	3.400170E-42	ukb-b-19953	Body mass index (BMI)
rs10505836	0.01848510	0.00876151	ieu-b-18	0.0229471	115803	0.7026000	Multiple Sclerosis	461460	0.00287086	1.200000E-10	ukb-b-19953	Body mass index (BMI)
rs10510025	0.01756430	-0.00419120	ieu-b-18	0.0187046	115803	0.8227000	Multiple Sclerosis	461460	0.00229886	2.199890E-14	ukb-b-19953	Body mass index (BMI)
rs1064213	0.01493010	-0.01222500	ieu-b-18	0.0162836	115803	0.4528000	Multiple Sclerosis	461460	0.00197196	3.699990E-14	ukb-b-19953	Body mass index (BMI)
rs10742752	0.01179790	-0.00319489	ieu-b-18	0.0168481	115803	0.8496000	Multiple Sclerosis	461460	0.00202961	6.100000E-09	ukb-b-19953	Body mass index (BMI)
rs10756714	-0.02078620	-0.01685710	ieu-b-18	0.0166318	115803	0.3108000	Multiple Sclerosis	461460	0.00199408	1.900200E-25	ukb-b-19953	Body mass index (BMI)
rs10756792	-0.01905820	-0.01045450	ieu-b-18	0.0185385	115803	0.5728000	Multiple Sclerosis	461460	0.00227201	4.900040E-17	ukb-b-19953	Body mass index (BMI)
rs10760277	0.01387340	-0.02790700	ieu-b-18	0.017061	115803	0.1019000	Multiple Sclerosis	461460	0.00203825	1.000000E-11	ukb-b-19953	Body mass index (BMI)
rs10780248	-0.01212320	-0.01025240	ieu-b-18	0.0163174	115803	0.5298000	Multiple Sclerosis	461460	0.00199415	1.200000E-09	ukb-b-19953	Body mass index (BMI)
rs1078141	0.01422390	-0.01065660	ieu-b-18	0.0169195	115803	0.5287990	Multiple Sclerosis	461460	0.00205862	4.900040E-12	ukb-b-19953	Body mass index (BMI)
rs10799778	-0.01822370	0.02583080	ieu-b-18	0.0221855	115803	0.2443000	Multiple Sclerosis	461460	0.00264876	6.000670E-12	ukb-b-19953	Body mass index (BMI)
rs10809621	-0.01253050	-0.00100050	ieu-b-18	0.0174202	115803	0.9542000	Multiple Sclerosis	461460	0.00207043	1.400010E-09	ukb-b-19953	Body mass index (BMI)
rs10824211	0.02076880	0.01607020	ieu-b-18	0.0248658	115803	0.5181000	Multiple Sclerosis	461460	0.00286631	4.300310E-13	ukb-b-19953	Body mass index (BMI)
rs10832778	0.01156280	0.02420470	ieu-b-18	0.0167895	115803	0.1494000	Multiple Sclerosis	461460	0.00203985	1.400010E-08	ukb-b-19953	Body mass index (BMI)
rs10887578	0.01332120	0.03448790	ieu-b-18	0.0166709	115803	0.0385700	Multiple Sclerosis	461460	0.00198909	2.099910E-11	ukb-b-19953	Body mass index (BMI)
rs10927006	-0.01681560	0.00190181	ieu-b-18	0.0237961	115803	0.9363000	Multiple Sclerosis	461460	0.00281467	2.300010E-09	ukb-b-19953	Body mass index (BMI)
rs10965698	-0.01127410	-0.00924258	ieu-b-18	0.0168888	115803	0.5842010	Multiple Sclerosis	461460	0.002052	3.899960E-08	ukb-b-19953	Body mass index (BMI)
rs10989067	0.01695140	0.02521520	ieu-b-18	0.0171506	115803	0.1415000	Multiple Sclerosis	461460	0.00212349	1.399910E-15	ukb-b-19953	Body mass index (BMI)
rs11001963	0.01167940	-0.01350830	ieu-b-18	0.0164098	115803	0.4104000	Multiple Sclerosis	461460	0.00201921	7.299950E-09	ukb-b-19953	Body mass index (BMI)
rs11009685	-0.01308340	0.02829660	ieu-b-18	0.0199895	115803	0.1569000	Multiple Sclerosis	461460	0.00230766	1.400010E-08	ukb-b-19953	Body mass index (BMI)
rs11012732	0.02164250	0.03407400	ieu-b-18	0.0176122	115803	0.0530298	Multiple Sclerosis	461460	0.00210126	7.100680E-25	ukb-b-19953	Body mass index (BMI)
rs11079849	-0.02009300	0.00369317	ieu-b-18	0.0185393	115803	0.8421000	Multiple Sclerosis	461460	0.00211163	1.800110E-21	ukb-b-19953	Body mass index (BMI)
rs11099020	-0.01420380	0.01429730	ieu-b-18	0.0172757	115803	0.4079000	Multiple Sclerosis	461460	0.00206202	5.600150E-12	ukb-b-19953	Body mass index (BMI)
rs11115160	-0.01307680	-0.00239712	ieu-b-18	0.0193286	115803	0.9013000	Multiple Sclerosis	461460	0.00233641	2.199990E-08	ukb-b-19953	Body mass index (BMI)
rs11122450	-0.01163160	0.01369330	ieu-b-18	0.0166879	115803	0.4119000	Multiple Sclerosis	461460	0.00202425	9.099970E-09	ukb-b-19953	Body mass index (BMI)
rs11134679	0.01824590	0.01004930	ieu-b-18	0.0174685	115803	0.5651000	Multiple Sclerosis	461460	0.00213286	1.200050E-17	ukb-b-19953	Body mass index (BMI)
rs11150745	-0.02116110	-0.06335030	ieu-b-18	0.0177427	115803	0.0003563	Multiple Sclerosis	461460	0.00212961	2.900010E-23	ukb-b-19953	Body mass index (BMI)
rs11165643	0.01933190	0.01724790	ieu-b-18	0.0164975	115803	0.2958000	Multiple Sclerosis	461460	0.00200337	4.900040E-22	ukb-b-19953	Body mass index (BMI)
rs111689389	-0.01367060	0.00924258	ieu-b-18	0.0180847	115803	0.6093000	Multiple Sclerosis	461460	0.00219522	4.700020E-10	ukb-b-19953	Body mass index (BMI)
rs11218510	-0.01446160	-0.01350830	ieu-b-18	0.0169142	115803	0.4245000	Multiple Sclerosis	461460	0.00202121	8.400400E-13	ukb-b-19953	Body mass index (BMI)
rs11250094	-0.02029070	-0.00259663	ieu-b-18	0.0168877	115803	0.8778000	Multiple Sclerosis	461460	0.00199209	2.299850E-24	ukb-b-19953	Body mass index (BMI)
rs1126930	0.03226950	0.01999860	ieu-b-18	0.112656	115803	0.8591000	Multiple Sclerosis	461460	0.00535892	1.700000E-09	ukb-b-19953	Body mass index (BMI)
rs113603865	0.01860130	0.01389610	ieu-b-18	0.0200424	115803	0.4881000	Multiple Sclerosis	461460	0.00242689	1.800110E-14	ukb-b-19953	Body mass index (BMI)
rs113624107	0.01504610	0.05213580	ieu-b-18	0.0195935	115803	0.0077940	Multiple Sclerosis	461460	0.00236876	2.100000E-10	ukb-b-19953	Body mass index (BMI)
rs11525873	-0.02397810	0.06432520	ieu-b-18	0.0282798	115803	0.0229298	Multiple Sclerosis	461460	0.00333603	6.599330E-13	ukb-b-19953	Body mass index (BMI)
rs11607476	0.01571200	0.04730130	ieu-b-18	0.0166816	115803	0.0045750	Multiple Sclerosis	461460	0.00199179	3.100270E-15	ukb-b-19953	Body mass index (BMI)
rs11610621	0.01648690	-0.02088050	ieu-b-18	0.0241068	115803	0.3864000	Multiple Sclerosis	461460	0.00278318	3.099990E-09	ukb-b-19953	Body mass index (BMI)
rs11630647	-0.01267400	-0.00866237	ieu-b-18	0.018899	115803	0.6467000	Multiple Sclerosis	461460	0.00228082	2.699980E-08	ukb-b-19953	Body mass index (BMI)
rs11634851	0.01186710	0.01989660	ieu-b-18	0.0162641	115803	0.2212000	Multiple Sclerosis	461460	0.00198456	2.199990E-09	ukb-b-19953	Body mass index (BMI)
rs11642090	0.01141840	0.02142790	ieu-b-18	0.0175083	115803	0.2210000	Multiple Sclerosis	461460	0.00205925	2.900010E-08	ukb-b-19953	Body mass index (BMI)
rs11656076	-0.01542850	-0.02576520	ieu-b-18	0.0193494	115803	0.1830000	Multiple Sclerosis	461460	0.00237054	7.599760E-11	ukb-b-19953	Body mass index (BMI)
rs1167311	-0.01926490	0.00279609	ieu-b-18	0.0171782	115803	0.8707000	Multiple Sclerosis	461460	0.00213109	1.599930E-19	ukb-b-19953	Body mass index (BMI)
rs11675464	0.01197510	0.05266270	ieu-b-18	0.01651	115803	0.0014240	Multiple Sclerosis	461460	0.00198558	1.600000E-09	ukb-b-19953	Body mass index (BMI)
rs11691869	-0.01931580	0.02153010	ieu-b-18	0.0181682	115803	0.2360000	Multiple Sclerosis	461460	0.00205562	5.600150E-21	ukb-b-19953	Body mass index (BMI)
rs11699828	-0.03354180	0.24219900	ieu-b-18	0.182637	115803	0.1848000	Multiple Sclerosis	461460	0.00582677	8.600030E-09	ukb-b-19953	Body mass index (BMI)
rs11709402	0.02281920	-0.00578324	ieu-b-18	0.0182784	115803	0.7517010	Multiple Sclerosis	461460	0.00220796	4.900040E-25	ukb-b-19953	Body mass index (BMI)
rs11757278	-0.01462690	-0.01257880	ieu-b-18	0.0175844	115803	0.4744000	Multiple Sclerosis	461460	0.00214834	9.899200E-12	ukb-b-19953	Body mass index (BMI)
rs11778219	0.01579600	0.01359200	ieu-b-18	0.0221086	115803	0.5387000	Multiple Sclerosis	461460	0.0026874	4.200010E-09	ukb-b-19953	Body mass index (BMI)
rs11919665	-0.01278540	-0.01136430	ieu-b-18	0.0178565	115803	0.5244990	Multiple Sclerosis	461460	0.00211292	1.400010E-09	ukb-b-19953	Body mass index (BMI)
rs12001437	0.01218360	0.00030005	ieu-b-18	0.0174737	115803	0.9863000	Multiple Sclerosis	461460	0.00205069	2.800010E-09	ukb-b-19953	Body mass index (BMI)
rs12072739	0.01570080	-0.03033520	ieu-b-18	0.0192945	115803	0.1159000	Multiple Sclerosis	461460	0.00236691	3.299890E-11	ukb-b-19953	Body mass index (BMI)
rs12088284	0.01393170	-0.01597180	ieu-b-18	0.0179734	115803	0.3742000	Multiple Sclerosis	461460	0.00214854	8.900200E-11	ukb-b-19953	Body mass index (BMI)
rs12089815	-0.01231210	-0.02347340	ieu-b-18	0.0182594	115803	0.1986000	Multiple Sclerosis	461460	0.00198606	5.699940E-10	ukb-b-19953	Body mass index (BMI)
rs12140153	-0.03307500	0.02030480	ieu-b-18	0.0440852	115803	0.6451000	Multiple Sclerosis	461460	0.0034589	1.200050E-21	ukb-b-19953	Body mass index (BMI)
rs12149660	-0.02273380	-0.00468899	ieu-b-18	0.0253059	115803	0.8530000	Multiple Sclerosis	461460	0.00311586	2.999850E-13	ukb-b-19953	Body mass index (BMI)
rs12259464	0.01308950	0.00702462	ieu-b-18	0.0162018	115803	0.6645990	Multiple Sclerosis	461460	0.00198686	4.499870E-11	ukb-b-19953	Body mass index (BMI)
rs12273545	0.02483930	-0.09676370	ieu-b-18	0.0358999	115803	0.0070311	Multiple Sclerosis	461460	0.00428551	6.800020E-09	ukb-b-19953	Body mass index (BMI)
rs1229984	0.03735700	0.02244620	ieu-b-18	0.0719003	115803	0.7548990	Multiple Sclerosis	461460	0.00599385	4.600020E-10	ukb-b-19953	Body mass index (BMI)
rs12364470	0.01927050	-0.02430230	ieu-b-18	0.0230198	115803	0.2911000	Multiple Sclerosis	461460	0.0026658	4.900040E-13	ukb-b-19953	Body mass index (BMI)
rs12440603	0.01392850	0.01439590	ieu-b-18	0.0164845	115803	0.3825000	Multiple Sclerosis	461460	0.0020011	3.400170E-12	ukb-b-19953	Body mass index (BMI)
rs12459368	-0.01701410	-0.01990070	ieu-b-18	0.0189641	115803	0.2940000	Multiple Sclerosis	461460	0.00223235	2.499770E-14	ukb-b-19953	Body mass index (BMI)
rs12507026	0.02898960	0.02367810	ieu-b-18	0.0164566	115803	0.1502000	Multiple Sclerosis	461460	0.0019931	6.299410E-48	ukb-b-19953	Body mass index (BMI)
rs12541408	-0.01432610	-0.00050013	ieu-b-18	0.0166242	115803	0.9760000	Multiple Sclerosis	461460	0.00212723	1.599930E-11	ukb-b-19953	Body mass index (BMI)
rs1266874	0.01409660	0.01217380	ieu-b-18	0.0170723	115803	0.4758000	Multiple Sclerosis	461460	0.0020712	1.000000E-11	ukb-b-19953	Body mass index (BMI)
rs12681792	0.01486930	0.03687150	ieu-b-18	0.0219582	115803	0.0931194	Multiple Sclerosis	461460	0.00251693	3.500020E-09	ukb-b-19953	Body mass index (BMI)
rs12692596	0.01308720	-0.00419120	ieu-b-18	0.0169944	115803	0.8052000	Multiple Sclerosis	461460	0.00203738	1.299990E-10	ukb-b-19953	Body mass index (BMI)
rs12696039	-0.01528020	-0.00169856	ieu-b-18	0.0237979	115803	0.9431000	Multiple Sclerosis	461460	0.0027705	3.500020E-08	ukb-b-19953	Body mass index (BMI)
rs1286058	0.01491260	0.04955180	ieu-b-18	0.017966	115803	0.0058141	Multiple Sclerosis	461460	0.00216922	6.200120E-12	ukb-b-19953	Body mass index (BMI)
rs12881629	0.02207480	0.00260339	ieu-b-18	0.0400722	115803	0.9482000	Multiple Sclerosis	461460	0.00358929	7.699990E-10	ukb-b-19953	Body mass index (BMI)
rs12921986	0.02033270	-0.00935609	ieu-b-18	0.0352009	115803	0.7904000	Multiple Sclerosis	461460	0.0036983	3.799970E-08	ukb-b-19953	Body mass index (BMI)
rs12937411	-0.01718700	-0.07224820	ieu-b-18	0.0165918	115803	0.0000133	Multiple Sclerosis	461460	0.00201383	1.399910E-17	ukb-b-19953	Body mass index (BMI)
rs1296328	-0.01886200	-0.00029996	ieu-b-18	0.0154396	115803	0.9845000	Multiple Sclerosis	461460	0.001999	3.900320E-21	ukb-b-19953	Body mass index (BMI)
rs12974458	0.01524660	0.01754520	ieu-b-18	0.0175629	115803	0.3178000	Multiple Sclerosis	461460	0.00199717	2.299850E-14	ukb-b-19953	Body mass index (BMI)
rs13012070	-0.01365010	0.01460620	ieu-b-18	0.0194984	115803	0.4537990	Multiple Sclerosis	461460	0.00234795	6.100000E-09	ukb-b-19953	Body mass index (BMI)
rs13033310	0.01261100	-0.01146550	ieu-b-18	0.0186405	115803	0.5385000	Multiple Sclerosis	461460	0.0022826	3.299970E-08	ukb-b-19953	Body mass index (BMI)
rs13097918	0.01455610	0.02518030	ieu-b-18	0.0197357	115803	0.2020000	Multiple Sclerosis	461460	0.0024161	1.700000E-09	ukb-b-19953	Body mass index (BMI)
rs13107325	0.04757990	0.11350500	ieu-b-18	0.0378848	115803	0.0027350	Multiple Sclerosis	461460	0.00375479	8.499630E-37	ukb-b-19953	Body mass index (BMI)
rs13176429	0.01415550	-0.04046990	ieu-b-18	0.0176443	115803	0.0218102	Multiple Sclerosis	461460	0.00213122	3.100270E-11	ukb-b-19953	Body mass index (BMI)
rs1320251	-0.01803210	0.01281750	ieu-b-18	0.0169488	115803	0.4495000	Multiple Sclerosis	461460	0.00199407	1.500030E-19	ukb-b-19953	Body mass index (BMI)
rs13218383	-0.01440250	-0.02800420	ieu-b-18	0.0170805	115803	0.1011000	Multiple Sclerosis	461460	0.00209236	5.799630E-12	ukb-b-19953	Body mass index (BMI)
rs1322842	-0.01312920	0.00189820	ieu-b-18	0.017954	115803	0.9158000	Multiple Sclerosis	461460	0.00203539	1.099990E-10	ukb-b-19953	Body mass index (BMI)
rs13248187	0.01576400	0.01065660	ieu-b-18	0.0186101	115803	0.5668990	Multiple Sclerosis	461460	0.00224155	1.999860E-12	ukb-b-19953	Body mass index (BMI)
rs1327259	-0.01485320	0.00110061	ieu-b-18	0.0164326	115803	0.9466000	Multiple Sclerosis	461460	0.00203363	2.800270E-13	ukb-b-19953	Body mass index (BMI)
rs13291723	0.01103180	0.00200200	ieu-b-18	0.0165643	115803	0.9038000	Multiple Sclerosis	461460	0.00199876	3.400010E-08	ukb-b-19953	Body mass index (BMI)
rs1330199	-0.01175090	0.01301490	ieu-b-18	0.0162367	115803	0.4228000	Multiple Sclerosis	461460	0.00198642	3.299970E-09	ukb-b-19953	Body mass index (BMI)
rs13420048	-0.01548170	0.00300451	ieu-b-18	0.0169736	115803	0.8595000	Multiple Sclerosis	461460	0.00205272	4.600450E-14	ukb-b-19953	Body mass index (BMI)
rs13427822	-0.01815150	-0.01281750	ieu-b-18	0.0183557	115803	0.4850000	Multiple Sclerosis	461460	0.00224142	5.600150E-16	ukb-b-19953	Body mass index (BMI)
rs1346841	-0.01305600	-0.01106090	ieu-b-18	0.016745	115803	0.5088990	Multiple Sclerosis	461460	0.0020172	9.700630E-11	ukb-b-19953	Body mass index (BMI)
rs1360201	0.01300750	0.02122360	ieu-b-18	0.0161722	115803	0.1894000	Multiple Sclerosis	461460	0.00197747	4.799540E-11	ukb-b-19953	Body mass index (BMI)
rs13642	-0.01613630	0.00631993	ieu-b-18	0.0169895	115803	0.7099000	Multiple Sclerosis	461460	0.00205695	4.300310E-15	ukb-b-19953	Body mass index (BMI)
rs1438945	-0.01338240	-0.01247750	ieu-b-18	0.0185381	115803	0.5009000	Multiple Sclerosis	461460	0.00219791	1.099990E-09	ukb-b-19953	Body mass index (BMI)
rs1441264	0.01790330	-0.00360650	ieu-b-18	0.0170937	115803	0.8329000	Multiple Sclerosis	461460	0.00205854	3.400170E-18	ukb-b-19953	Body mass index (BMI)
rs1451963	0.02220100	-0.00707491	ieu-b-18	0.0308482	115803	0.8186000	Multiple Sclerosis	461460	0.00360585	7.399970E-10	ukb-b-19953	Body mass index (BMI)
rs1454687	-0.02079160	-0.01126320	ieu-b-18	0.0163199	115803	0.4901000	Multiple Sclerosis	461460	0.00197396	6.099580E-26	ukb-b-19953	Body mass index (BMI)
rs1458156	0.01407500	0.01460620	ieu-b-18	0.0163338	115803	0.3712000	Multiple Sclerosis	461460	0.00197951	1.200050E-12	ukb-b-19953	Body mass index (BMI)
rs146569428	0.01395330	-0.00169856	ieu-b-18	0.0214214	115803	0.9368000	Multiple Sclerosis	461460	0.00248571	2.000000E-08	ukb-b-19953	Body mass index (BMI)
rs1471093	0.01346190	0.04322070	ieu-b-18	0.0171336	115803	0.0116501	Multiple Sclerosis	461460	0.00203996	4.100150E-11	ukb-b-19953	Body mass index (BMI)
rs1471740	0.01935760	0.00974735	ieu-b-18	0.0187986	115803	0.6041000	Multiple Sclerosis	461460	0.00225404	8.900200E-18	ukb-b-19953	Body mass index (BMI)
rs147568678	-0.01333270	0.00090041	ieu-b-18	0.0199493	115803	0.9640000	Multiple Sclerosis	461460	0.00233034	1.099990E-08	ukb-b-19953	Body mass index (BMI)
rs1477290	0.03377720	0.00816656	ieu-b-18	0.023303	115803	0.7260010	Multiple Sclerosis	461460	0.00289815	2.199890E-31	ukb-b-19953	Body mass index (BMI)
rs1503526	0.01543080	0.02955880	ieu-b-18	0.0162306	115803	0.0685804	Multiple Sclerosis	461460	0.00197681	5.900650E-15	ukb-b-19953	Body mass index (BMI)
rs156201	0.01318990	0.01656210	ieu-b-18	0.0191413	115803	0.3869000	Multiple Sclerosis	461460	0.00228932	8.300040E-09	ukb-b-19953	Body mass index (BMI)
rs156914	0.01115520	0.03440140	ieu-b-18	0.0237158	115803	0.1469000	Multiple Sclerosis	461460	0.00197308	1.600000E-08	ukb-b-19953	Body mass index (BMI)
rs1582931	-0.01334190	0.02029270	ieu-b-18	0.0166814	115803	0.2238000	Multiple Sclerosis	461460	0.0019956	2.299850E-11	ukb-b-19953	Body mass index (BMI)
rs1608113	-0.01177660	-0.00853633	ieu-b-18	0.0168628	115803	0.6127000	Multiple Sclerosis	461460	0.00205086	9.299940E-09	ukb-b-19953	Body mass index (BMI)
rs1609010	0.02097730	-0.01308520	ieu-b-18	0.0164234	115803	0.4256000	Multiple Sclerosis	461460	0.0019963	7.899510E-26	ukb-b-19953	Body mass index (BMI)
rs16916303	-0.01923690	0.01673930	ieu-b-18	0.0267652	115803	0.5317000	Multiple Sclerosis	461460	0.00307931	4.200010E-10	ukb-b-19953	Body mass index (BMI)
rs17056301	0.01358310	0.02878180	ieu-b-18	0.0186018	115803	0.1218000	Multiple Sclerosis	461460	0.0022697	2.199990E-09	ukb-b-19953	Body mass index (BMI)
rs17132130	-0.01784180	-0.00039992	ieu-b-18	0.0204532	115803	0.9844000	Multiple Sclerosis	461460	0.00238564	7.500670E-14	ukb-b-19953	Body mass index (BMI)
rs17149254	-0.02135070	-0.10524900	ieu-b-18	0.0602384	115803	0.0805991	Multiple Sclerosis	461460	0.00255804	7.000030E-17	ukb-b-19953	Body mass index (BMI)
rs17289010	-0.01346610	-0.01390290	ieu-b-18	0.0175177	115803	0.4274000	Multiple Sclerosis	461460	0.00210438	1.600000E-10	ukb-b-19953	Body mass index (BMI)
rs17399739	0.02707130	0.04793050	ieu-b-18	0.0323385	115803	0.1383000	Multiple Sclerosis	461460	0.00391006	4.400480E-12	ukb-b-19953	Body mass index (BMI)
rs17446299	0.01532350	0.00320513	ieu-b-18	0.0222774	115803	0.8856000	Multiple Sclerosis	461460	0.00266515	8.900000E-09	ukb-b-19953	Body mass index (BMI)
rs17544384	0.01409310	-0.00727348	ieu-b-18	0.0201482	115803	0.7181000	Multiple Sclerosis	461460	0.00241434	5.300050E-09	ukb-b-19953	Body mass index (BMI)
rs17668356	-0.02305430	0.01271880	ieu-b-18	0.0234778	115803	0.5880000	Multiple Sclerosis	461460	0.00279318	1.500030E-16	ukb-b-19953	Body mass index (BMI)
rs17770336	0.02429310	0.01887710	ieu-b-18	0.0174918	115803	0.2805000	Multiple Sclerosis	461460	0.00211161	1.299870E-30	ukb-b-19953	Body mass index (BMI)
rs1778830	0.01407750	0.03190360	ieu-b-18	0.0169535	115803	0.0598605	Multiple Sclerosis	461460	0.00205596	7.500670E-12	ukb-b-19953	Body mass index (BMI)
rs1788808	-0.02040400	0.01024730	ieu-b-18	0.0162461	115803	0.5282000	Multiple Sclerosis	461460	0.00198255	7.700160E-25	ukb-b-19953	Body mass index (BMI)
rs1793636	-0.01333430	-0.01656210	ieu-b-18	0.0179398	115803	0.3559000	Multiple Sclerosis	461460	0.0021408	4.700020E-10	ukb-b-19953	Body mass index (BMI)
rs1805123	-0.01675690	0.00511305	ieu-b-18	0.0189866	115803	0.7877000	Multiple Sclerosis	461460	0.00229711	2.999850E-13	ukb-b-19953	Body mass index (BMI)
rs1834144	-0.01401120	0.00935609	ieu-b-18	0.0173355	115803	0.5894000	Multiple Sclerosis	461460	0.00205213	8.600030E-12	ukb-b-19953	Body mass index (BMI)
rs1860750	0.01170380	0.00330546	ieu-b-18	0.0164872	115803	0.8411000	Multiple Sclerosis	461460	0.00198192	3.500020E-09	ukb-b-19953	Body mass index (BMI)
rs1861410	-0.02125580	-0.01024730	ieu-b-18	0.016453	115803	0.5334000	Multiple Sclerosis	461460	0.00198918	1.200050E-26	ukb-b-19953	Body mass index (BMI)
rs1884897	0.02000100	0.01103880	ieu-b-18	0.0168106	115803	0.5114000	Multiple Sclerosis	461460	0.00205634	2.299850E-22	ukb-b-19953	Body mass index (BMI)
rs1919243	0.01167590	-0.03864360	ieu-b-18	0.0171486	115803	0.0242298	Multiple Sclerosis	461460	0.00200175	5.499970E-09	ukb-b-19953	Body mass index (BMI)
rs1967772	-0.01704340	-0.03275760	ieu-b-18	0.0183382	115803	0.0740508	Multiple Sclerosis	461460	0.00220377	1.000000E-14	ukb-b-19953	Body mass index (BMI)
rs2035936	0.03708630	0.05435060	ieu-b-18	0.0388854	115803	0.1622000	Multiple Sclerosis	461460	0.00435778	1.699810E-17	ukb-b-19953	Body mass index (BMI)
rs2051559	0.02040780	-0.02673930	ieu-b-18	0.0245002	115803	0.2751000	Multiple Sclerosis	461460	0.00291963	2.800270E-12	ukb-b-19953	Body mass index (BMI)
rs2075466	0.01329030	-0.02156580	ieu-b-18	0.0186602	115803	0.2478000	Multiple Sclerosis	461460	0.00223627	2.800010E-09	ukb-b-19953	Body mass index (BMI)
rs2102278	0.01185830	0.03295110	ieu-b-18	0.0183717	115803	0.0728803	Multiple Sclerosis	461460	0.0021139	2.000000E-08	ukb-b-19953	Body mass index (BMI)
rs2133561	-0.01409700	-0.01694270	ieu-b-18	0.0184324	115803	0.3580000	Multiple Sclerosis	461460	0.00204744	5.799630E-12	ukb-b-19953	Body mass index (BMI)
rs213518	0.01578940	0.06038710	ieu-b-18	0.0233638	115803	0.0097481	Multiple Sclerosis	461460	0.00280491	1.799990E-08	ukb-b-19953	Body mass index (BMI)
rs2153740	-0.01125510	-0.04322070	ieu-b-18	0.0167285	115803	0.0097760	Multiple Sclerosis	461460	0.00199313	1.600000E-08	ukb-b-19953	Body mass index (BMI)
rs215634	-0.01552230	-0.01093990	ieu-b-18	0.0166797	115803	0.5119000	Multiple Sclerosis	461460	0.00203492	2.399940E-14	ukb-b-19953	Body mass index (BMI)
rs2172131	-0.01493820	-0.00803217	ieu-b-18	0.0164	115803	0.6243000	Multiple Sclerosis	461460	0.00200386	8.999120E-14	ukb-b-19953	Body mass index (BMI)
rs217672	0.01701550	0.00611868	ieu-b-18	0.0189824	115803	0.7472000	Multiple Sclerosis	461460	0.00223046	2.399940E-14	ukb-b-19953	Body mass index (BMI)
rs2192158	-0.01501200	-0.01197140	ieu-b-18	0.0163591	115803	0.4643000	Multiple Sclerosis	461460	0.00198302	3.699990E-14	ukb-b-19953	Body mass index (BMI)
rs2216931	0.01691090	0.02537530	ieu-b-18	0.0173634	115803	0.1439000	Multiple Sclerosis	461460	0.00208587	5.199960E-16	ukb-b-19953	Body mass index (BMI)
rs2234458	-0.02038410	0.03988490	ieu-b-18	0.0175644	115803	0.0231601	Multiple Sclerosis	461460	0.00205579	3.599980E-23	ukb-b-19953	Body mass index (BMI)
rs2248551	0.01465230	0.03076850	ieu-b-18	0.0224017	115803	0.1696000	Multiple Sclerosis	461460	0.00266497	3.799970E-08	ukb-b-19953	Body mass index (BMI)
rs2253310	0.01732130	-0.00642057	ieu-b-18	0.0167568	115803	0.7016010	Multiple Sclerosis	461460	0.00204115	2.099910E-17	ukb-b-19953	Body mass index (BMI)
rs2289379	-0.01527650	0.01774170	ieu-b-18	0.0174108	115803	0.3082000	Multiple Sclerosis	461460	0.00202952	5.199960E-14	ukb-b-19953	Body mass index (BMI)
rs2307111	-0.02800420	0.01663760	ieu-b-18	0.0167892	115803	0.3217000	Multiple Sclerosis	461460	0.00202203	1.299870E-43	ukb-b-19953	Body mass index (BMI)
rs2342892	-0.01269720	0.01656210	ieu-b-18	0.0164288	115803	0.3134000	Multiple Sclerosis	461460	0.00197715	1.299990E-10	ukb-b-19953	Body mass index (BMI)
rs2381404	0.01396640	-0.00697561	ieu-b-18	0.0191175	115803	0.7152010	Multiple Sclerosis	461460	0.00229974	1.299990E-09	ukb-b-19953	Body mass index (BMI)
rs2383377	0.01612870	0.04416090	ieu-b-18	0.0263146	115803	0.0933104	Multiple Sclerosis	461460	0.00293564	3.899960E-08	ukb-b-19953	Body mass index (BMI)
rs2396625	-0.01891900	-0.02078250	ieu-b-18	0.0166224	115803	0.2112000	Multiple Sclerosis	461460	0.00201335	5.600150E-21	ukb-b-19953	Body mass index (BMI)
rs2398861	0.01799320	0.01877520	ieu-b-18	0.0185856	115803	0.3124000	Multiple Sclerosis	461460	0.00226733	2.099910E-15	ukb-b-19953	Body mass index (BMI)
rs2425816	0.01222840	0.04019720	ieu-b-18	0.0165177	115803	0.0149500	Multiple Sclerosis	461460	0.00201106	1.200000E-09	ukb-b-19953	Body mass index (BMI)
rs2433733	-0.01718050	0.00803217	ieu-b-18	0.0172253	115803	0.6410000	Multiple Sclerosis	461460	0.00210907	3.800140E-16	ukb-b-19953	Body mass index (BMI)
rs2439823	0.01919980	0.03440140	ieu-b-18	0.0165004	115803	0.0370800	Multiple Sclerosis	461460	0.00199108	5.300290E-22	ukb-b-19953	Body mass index (BMI)
rs2482356	-0.01135110	0.00190181	ieu-b-18	0.0161919	115803	0.9065000	Multiple Sclerosis	461460	0.00199506	1.299990E-08	ukb-b-19953	Body mass index (BMI)
rs2512892	0.01294680	0.00893984	ieu-b-18	0.0164625	115803	0.5871000	Multiple Sclerosis	461460	0.00199819	9.200260E-11	ukb-b-19953	Body mass index (BMI)
rs252761	-0.01150460	0.02508280	ieu-b-18	0.0168889	115803	0.1375000	Multiple Sclerosis	461460	0.00201769	1.200000E-08	ukb-b-19953	Body mass index (BMI)
rs2568958	0.02229280	0.00541463	ieu-b-18	0.0173883	115803	0.7554990	Multiple Sclerosis	461460	0.00201204	1.599930E-28	ukb-b-19953	Body mass index (BMI)
rs2569993	0.01267000	0.00945516	ieu-b-18	0.0175097	115803	0.5892000	Multiple Sclerosis	461460	0.00212202	2.399990E-09	ukb-b-19953	Body mass index (BMI)
rs2606228	-0.01387910	0.00621930	ieu-b-18	0.0173114	115803	0.7193990	Multiple Sclerosis	461460	0.00208379	2.700230E-11	ukb-b-19953	Body mass index (BMI)
rs2616143	-0.01387380	-0.00080032	ieu-b-18	0.0169317	115803	0.9623000	Multiple Sclerosis	461460	0.00212547	6.700390E-11	ukb-b-19953	Body mass index (BMI)
rs2618039	0.01439630	-0.02296160	ieu-b-18	0.0167373	115803	0.1701000	Multiple Sclerosis	461460	0.0020317	1.399910E-12	ukb-b-19953	Body mass index (BMI)
rs2678204	0.02416100	-0.00498754	ieu-b-18	0.0177155	115803	0.7783000	Multiple Sclerosis	461460	0.00208158	3.800140E-31	ukb-b-19953	Body mass index (BMI)
rs2725371	-0.01603010	-0.00611868	ieu-b-18	0.0179781	115803	0.7335990	Multiple Sclerosis	461460	0.00215845	1.100020E-13	ukb-b-19953	Body mass index (BMI)
rs2791643	-0.01338370	0.00200200	ieu-b-18	0.0190952	115803	0.9165000	Multiple Sclerosis	461460	0.00231332	7.199960E-09	ukb-b-19953	Body mass index (BMI)
rs28350	-0.01803350	-0.00722605	ieu-b-18	0.0211493	115803	0.7326000	Multiple Sclerosis	461460	0.00258234	2.900010E-12	ukb-b-19953	Body mass index (BMI)
rs28366156	-0.02648260	-0.14954000	ieu-b-18	0.0266736	115803	0.0000000	Multiple Sclerosis	461460	0.00292978	1.599930E-19	ukb-b-19953	Body mass index (BMI)
rs2837996	0.01266470	0.02917040	ieu-b-18	0.0170233	115803	0.0866104	Multiple Sclerosis	461460	0.00207859	1.099990E-09	ukb-b-19953	Body mass index (BMI)
rs28404639	-0.01171290	-0.01025240	ieu-b-18	0.0174523	115803	0.5569000	Multiple Sclerosis	461460	0.00205533	1.200000E-08	ukb-b-19953	Body mass index (BMI)
rs28489620	-0.01537370	0.00451016	ieu-b-18	0.0198997	115803	0.8207000	Multiple Sclerosis	461460	0.00220025	2.800270E-12	ukb-b-19953	Body mass index (BMI)
rs28568418	-0.01828250	-0.00707491	ieu-b-18	0.0263734	115803	0.7885010	Multiple Sclerosis	461460	0.00320812	1.200000E-08	ukb-b-19953	Body mass index (BMI)
rs2861685	-0.01712740	-0.01192860	ieu-b-18	0.0166002	115803	0.4724000	Multiple Sclerosis	461460	0.00199763	1.000000E-17	ukb-b-19953	Body mass index (BMI)
rs28670671	-0.01246380	0.00109940	ieu-b-18	0.0257162	115803	0.9659000	Multiple Sclerosis	461460	0.00226426	3.699990E-08	ukb-b-19953	Body mass index (BMI)
rs2870111	-0.01571510	0.00280393	ieu-b-18	0.0164678	115803	0.8648000	Multiple Sclerosis	461460	0.00201912	7.100680E-15	ukb-b-19953	Body mass index (BMI)
rs2875762	0.01533900	0.00039992	ieu-b-18	0.0185503	115803	0.9828000	Multiple Sclerosis	461460	0.0023121	3.299890E-11	ukb-b-19953	Body mass index (BMI)
rs2899644	0.01496590	0.03091710	ieu-b-18	0.0194103	115803	0.1112000	Multiple Sclerosis	461460	0.0023609	2.300010E-10	ukb-b-19953	Body mass index (BMI)
rs2920503	-0.01403070	-0.01084100	ieu-b-18	0.0184356	115803	0.5565000	Multiple Sclerosis	461460	0.00219666	1.700000E-10	ukb-b-19953	Body mass index (BMI)
rs2962334	0.04325520	0.03842900	ieu-b-18	0.0513575	115803	0.4543000	Multiple Sclerosis	461460	0.00703547	7.799920E-10	ukb-b-19953	Body mass index (BMI)
rs317656	-0.01447630	-0.01349060	ieu-b-18	0.0179813	115803	0.4531000	Multiple Sclerosis	461460	0.00221303	6.099580E-11	ukb-b-19953	Body mass index (BMI)
rs3213943	-0.01794060	0.04353400	ieu-b-18	0.0295955	115803	0.1413000	Multiple Sclerosis	461460	0.00288163	4.799990E-10	ukb-b-19953	Body mass index (BMI)
rs32421	0.01323150	-0.00219758	ieu-b-18	0.019441	115803	0.9100000	Multiple Sclerosis	461460	0.00237757	2.599980E-08	ukb-b-19953	Body mass index (BMI)
rs329118	-0.01657340	0.03448790	ieu-b-18	0.016582	115803	0.0375396	Multiple Sclerosis	461460	0.0020038	1.299870E-16	ukb-b-19953	Body mass index (BMI)
rs34045288	0.02347840	-0.00767051	ieu-b-18	0.0173873	115803	0.6591000	Multiple Sclerosis	461460	0.00209371	3.500260E-29	ukb-b-19953	Body mass index (BMI)
rs34153025	-0.03891510	-0.21171900	ieu-b-18	0.130265	115803	0.1041000	Multiple Sclerosis	461460	0.00678155	9.599970E-09	ukb-b-19953	Body mass index (BMI)
rs34234296	-0.01494700	-0.00816656	ieu-b-18	0.02093	115803	0.6964000	Multiple Sclerosis	461460	0.00204032	2.399940E-13	ukb-b-19953	Body mass index (BMI)
rs34481751	-0.01850290	-0.05439350	ieu-b-18	0.0236768	115803	0.0215998	Multiple Sclerosis	461460	0.00270404	7.800100E-12	ukb-b-19953	Body mass index (BMI)
rs34517439	0.03884800	0.11619700	ieu-b-18	0.0644786	115803	0.0715303	Multiple Sclerosis	461460	0.00304983	3.599980E-37	ukb-b-19953	Body mass index (BMI)
rs34696181	0.01143450	-0.00079968	ieu-b-18	0.017054	115803	0.9626000	Multiple Sclerosis	461460	0.00198283	8.100090E-09	ukb-b-19953	Body mass index (BMI)
rs347551	0.01386500	-0.01409890	ieu-b-18	0.0194183	115803	0.4678000	Multiple Sclerosis	461460	0.00201085	5.400080E-12	ukb-b-19953	Body mass index (BMI)
rs34811474	-0.02852930	-0.08038110	ieu-b-18	0.0422883	115803	0.0573297	Multiple Sclerosis	461460	0.00234269	4.100150E-34	ukb-b-19953	Body mass index (BMI)
rs349071	-0.01330620	-0.00863719	ieu-b-18	0.0166162	115803	0.6032000	Multiple Sclerosis	461460	0.00198231	1.900200E-11	ukb-b-19953	Body mass index (BMI)
rs35154326	-0.01304270	-0.03989360	ieu-b-18	0.019826	115803	0.0441998	Multiple Sclerosis	461460	0.00223442	5.300050E-09	ukb-b-19953	Body mass index (BMI)
rs35364449	0.02170690	-0.00538547	ieu-b-18	0.0324285	115803	0.8681000	Multiple Sclerosis	461460	0.00318335	9.200260E-12	ukb-b-19953	Body mass index (BMI)
rs355777	0.01527150	-0.01156660	ieu-b-18	0.0165076	115803	0.4834990	Multiple Sclerosis	461460	0.00201314	3.299890E-14	ukb-b-19953	Body mass index (BMI)
rs35697587	-0.01646770	-0.01075770	ieu-b-18	0.0162552	115803	0.5081000	Multiple Sclerosis	461460	0.00198067	9.200260E-17	ukb-b-19953	Body mass index (BMI)
rs35697691	0.02303050	0.00568382	ieu-b-18	0.0253223	115803	0.8224000	Multiple Sclerosis	461460	0.00352223	6.200120E-11	ukb-b-19953	Body mass index (BMI)
rs35809007	-0.01710090	-0.02839310	ieu-b-18	0.0172541	115803	0.0998504	Multiple Sclerosis	461460	0.00205627	9.099130E-17	ukb-b-19953	Body mass index (BMI)
rs35957544	-0.01964290	0.01291620	ieu-b-18	0.0165632	115803	0.4355000	Multiple Sclerosis	461460	0.00200438	1.100020E-22	ukb-b-19953	Body mass index (BMI)
rs36007635	-0.02104500	-0.02019470	ieu-b-18	0.0239645	115803	0.3994000	Multiple Sclerosis	461460	0.00286788	2.199890E-13	ukb-b-19953	Body mass index (BMI)
rs36061954	0.01284450	-0.00069976	ieu-b-18	0.0164163	115803	0.9660000	Multiple Sclerosis	461460	0.00201928	2.000000E-10	ukb-b-19953	Body mass index (BMI)
rs3764625	-0.01177140	-0.01389610	ieu-b-18	0.0164796	115803	0.3991000	Multiple Sclerosis	461460	0.00201523	5.199960E-09	ukb-b-19953	Body mass index (BMI)
rs3784710	-0.02970670	0.02518030	ieu-b-18	0.019692	115803	0.2010000	Multiple Sclerosis	461460	0.00236042	2.499770E-36	ukb-b-19953	Body mass index (BMI)
rs3803286	-0.01864170	-0.09871600	ieu-b-18	0.016958	115803	0.0000000	Multiple Sclerosis	461460	0.00209828	6.400300E-19	ukb-b-19953	Body mass index (BMI)
rs3807566	-0.01207270	0.00924258	ieu-b-18	0.0163045	115803	0.5708000	Multiple Sclerosis	461460	0.00199583	1.500000E-09	ukb-b-19953	Body mass index (BMI)
rs3814883	0.02401070	0.06838590	ieu-b-18	0.0168257	115803	0.0000482	Multiple Sclerosis	461460	0.00198424	1.000000E-33	ukb-b-19953	Body mass index (BMI)
rs3845344	0.01636090	-0.00955421	ieu-b-18	0.016828	115803	0.5702000	Multiple Sclerosis	461460	0.00201927	5.400080E-16	ukb-b-19953	Body mass index (BMI)
rs3851998	-0.01360710	-0.00883895	ieu-b-18	0.018631	115803	0.6351990	Multiple Sclerosis	461460	0.00226874	2.000000E-09	ukb-b-19953	Body mass index (BMI)
rs3866805	0.01178350	0.01321230	ieu-b-18	0.0171176	115803	0.4402000	Multiple Sclerosis	461460	0.00206546	1.200000E-08	ukb-b-19953	Body mass index (BMI)
rs3897102	0.01208380	-0.03033520	ieu-b-18	0.0182855	115803	0.0971203	Multiple Sclerosis	461460	0.00202716	2.500000E-09	ukb-b-19953	Body mass index (BMI)
rs3901286	-0.02255530	0.03796990	ieu-b-18	0.0223792	115803	0.0897594	Multiple Sclerosis	461460	0.00275602	2.700230E-16	ukb-b-19953	Body mass index (BMI)
rs3902951	0.01410190	0.01379470	ieu-b-18	0.0193849	115803	0.4767000	Multiple Sclerosis	461460	0.00235267	2.000000E-09	ukb-b-19953	Body mass index (BMI)
rs3935190	-0.01448640	-0.00692391	ieu-b-18	0.0174009	115803	0.6906990	Multiple Sclerosis	461460	0.00199701	4.000370E-13	ukb-b-19953	Body mass index (BMI)
rs394608	0.01863530	0.00430927	ieu-b-18	0.0162686	115803	0.7910990	Multiple Sclerosis	461460	0.00199484	9.499480E-21	ukb-b-19953	Body mass index (BMI)
rs396755	-0.01236390	-0.02673930	ieu-b-18	0.016509	115803	0.1053000	Multiple Sclerosis	461460	0.00199937	6.299990E-10	ukb-b-19953	Body mass index (BMI)
rs40071	-0.02616660	-0.03642840	ieu-b-18	0.0210527	115803	0.0835699	Multiple Sclerosis	461460	0.00258163	3.800140E-24	ukb-b-19953	Body mass index (BMI)
rs4017425	-0.01258170	0.01136430	ieu-b-18	0.0161893	115803	0.4827000	Multiple Sclerosis	461460	0.00198035	2.100000E-10	ukb-b-19953	Body mass index (BMI)
rs4055791	-0.01780820	0.00069976	ieu-b-18	0.0157222	115803	0.9645000	Multiple Sclerosis	461460	0.00200801	7.399460E-19	ukb-b-19953	Body mass index (BMI)
rs406388	0.01597300	-0.03905270	ieu-b-18	0.0215718	115803	0.0702393	Multiple Sclerosis	461460	0.00260181	8.300040E-10	ukb-b-19953	Body mass index (BMI)
rs41279738	0.06842630	-0.04898060	ieu-b-18	0.0483061	115803	0.3106000	Multiple Sclerosis	461460	0.0062225	4.000370E-28	ukb-b-19953	Body mass index (BMI)
rs4148155	-0.02296810	0.00420884	ieu-b-18	0.0267119	115803	0.8748000	Multiple Sclerosis	461460	0.00310659	1.399910E-13	ukb-b-19953	Body mass index (BMI)
rs4261944	0.01385530	-0.00219758	ieu-b-18	0.0172625	115803	0.8987000	Multiple Sclerosis	461460	0.00205652	1.599930E-11	ukb-b-19953	Body mass index (BMI)
rs4267103	0.01539160	0.02742060	ieu-b-18	0.0222498	115803	0.2178000	Multiple Sclerosis	461460	0.00254312	1.400010E-09	ukb-b-19953	Body mass index (BMI)
rs4284600	0.01196730	-0.01035340	ieu-b-18	0.0164103	115803	0.5281000	Multiple Sclerosis	461460	0.00199506	2.000000E-09	ukb-b-19953	Body mass index (BMI)
rs429343	-0.01737950	-0.02460010	ieu-b-18	0.0165849	115803	0.1380000	Multiple Sclerosis	461460	0.00199729	3.299890E-18	ukb-b-19953	Body mass index (BMI)
rs429358	-0.02667230	-0.07445790	ieu-b-18	0.0247526	115803	0.0026290	Multiple Sclerosis	461460	0.00274373	2.399940E-22	ukb-b-19953	Body mass index (BMI)
rs4307239	0.01214130	-0.00853633	ieu-b-18	0.0168296	115803	0.6119990	Multiple Sclerosis	461460	0.00198816	1.000000E-09	ukb-b-19953	Body mass index (BMI)
rs4419475	0.01148700	-0.00279609	ieu-b-18	0.0168366	115803	0.8681000	Multiple Sclerosis	461460	0.0020106	1.099990E-08	ukb-b-19953	Body mass index (BMI)
rs4444317	-0.01616040	-0.00608147	ieu-b-18	0.0198396	115803	0.7592000	Multiple Sclerosis	461460	0.00242104	2.499770E-11	ukb-b-19953	Body mass index (BMI)
rs4456769	0.01461110	0.01340970	ieu-b-18	0.0172185	115803	0.4361000	Multiple Sclerosis	461460	0.00210138	3.599980E-12	ukb-b-19953	Body mass index (BMI)
rs4477562	0.02961180	0.00974735	ieu-b-18	0.0249554	115803	0.6961010	Multiple Sclerosis	461460	0.00298011	2.900010E-23	ukb-b-19953	Body mass index (BMI)
rs4482463	-0.03131750	-0.03428090	ieu-b-18	0.0308257	115803	0.2661000	Multiple Sclerosis	461460	0.00370692	2.999850E-17	ukb-b-19953	Body mass index (BMI)
rs45486197	0.02576600	0.02891400	ieu-b-18	0.0455527	115803	0.5256000	Multiple Sclerosis	461460	0.00403737	1.700000E-10	ukb-b-19953	Body mass index (BMI)
rs4605363	0.01636380	0.00974735	ieu-b-18	0.0174024	115803	0.5754000	Multiple Sclerosis	461460	0.00207733	3.299890E-15	ukb-b-19953	Body mass index (BMI)
rs4648450	-0.01483700	0.08359900	ieu-b-18	0.0166762	115803	0.0000005	Multiple Sclerosis	461460	0.00198775	8.400400E-14	ukb-b-19953	Body mass index (BMI)
rs4658403	-0.01888770	-0.00150113	ieu-b-18	0.0213704	115803	0.9440000	Multiple Sclerosis	461460	0.00264997	1.000000E-12	ukb-b-19953	Body mass index (BMI)
rs4672338	0.01356460	-0.00029996	ieu-b-18	0.0170941	115803	0.9860000	Multiple Sclerosis	461460	0.00208669	8.000180E-11	ukb-b-19953	Body mass index (BMI)
rs4722398	0.01869930	-0.01153320	ieu-b-18	0.024491	115803	0.6376990	Multiple Sclerosis	461460	0.00287545	7.899510E-11	ukb-b-19953	Body mass index (BMI)
rs4737188	-0.01244470	0.00571631	ieu-b-18	0.0164677	115803	0.7285010	Multiple Sclerosis	461460	0.00198274	3.500020E-10	ukb-b-19953	Body mass index (BMI)
rs4764949	-0.01839240	-0.00895974	ieu-b-18	0.017424	115803	0.6071000	Multiple Sclerosis	461460	0.00211197	3.100270E-18	ukb-b-19953	Body mass index (BMI)
rs4790292	-0.02545090	0.03355680	ieu-b-18	0.0240255	115803	0.1625000	Multiple Sclerosis	461460	0.00275603	2.600160E-20	ukb-b-19953	Body mass index (BMI)
rs4820410	-0.01774570	0.03988490	ieu-b-18	0.0171449	115803	0.0200000	Multiple Sclerosis	461460	0.00208527	1.699810E-17	ukb-b-19953	Body mass index (BMI)
rs4832298	-0.01596500	-0.01734860	ieu-b-18	0.0176375	115803	0.3253000	Multiple Sclerosis	461460	0.00212268	5.400080E-14	ukb-b-19953	Body mass index (BMI)
rs4858940	0.02292810	0.02955880	ieu-b-18	0.0268362	115803	0.2707000	Multiple Sclerosis	461460	0.00309976	1.399910E-13	ukb-b-19953	Body mass index (BMI)
rs4876611	0.01975490	0.02078250	ieu-b-18	0.0183385	115803	0.2571000	Multiple Sclerosis	461460	0.00220502	3.299890E-19	ukb-b-19953	Body mass index (BMI)
rs4929923	0.01894240	-0.02142790	ieu-b-18	0.0172213	115803	0.2134000	Multiple Sclerosis	461460	0.00206436	4.499870E-20	ukb-b-19953	Body mass index (BMI)
rs5011579	0.01402680	-0.00501254	ieu-b-18	0.019092	115803	0.7928990	Multiple Sclerosis	461460	0.00219322	1.600000E-10	ukb-b-19953	Body mass index (BMI)
rs512121	-0.01593590	0.03262650	ieu-b-18	0.0206436	115803	0.1140000	Multiple Sclerosis	461460	0.00252176	2.599980E-10	ukb-b-19953	Body mass index (BMI)
rs529200	0.01689650	0.01675880	ieu-b-18	0.0167273	115803	0.3164000	Multiple Sclerosis	461460	0.0019785	1.299870E-17	ukb-b-19953	Body mass index (BMI)
rs539515	0.04952910	0.00786896	ieu-b-18	0.0203505	115803	0.6990000	Multiple Sclerosis	461460	0.0024426	1.999860E-91	ukb-b-19953	Body mass index (BMI)
rs55714539	0.01757190	-0.11608400	ieu-b-18	0.0191464	115803	0.0000000	Multiple Sclerosis	461460	0.00210043	6.000670E-17	ukb-b-19953	Body mass index (BMI)
rs55726687	0.02483000	-0.00488803	ieu-b-18	0.0205401	115803	0.8119000	Multiple Sclerosis	461460	0.00242628	1.399910E-24	ukb-b-19953	Body mass index (BMI)
rs55769038	0.01609610	-0.00200200	ieu-b-18	0.0167215	115803	0.9047000	Multiple Sclerosis	461460	0.00201096	1.200050E-15	ukb-b-19953	Body mass index (BMI)
rs558887	-0.01299760	0.00521357	ieu-b-18	0.0182811	115803	0.7755000	Multiple Sclerosis	461460	0.00214881	1.500000E-09	ukb-b-19953	Body mass index (BMI)
rs559231	0.01349070	-0.00501254	ieu-b-18	0.0174962	115803	0.7744990	Multiple Sclerosis	461460	0.00203562	3.400170E-11	ukb-b-19953	Body mass index (BMI)
rs56038322	0.01392760	0.02142790	ieu-b-18	0.0204873	115803	0.2956000	Multiple Sclerosis	461460	0.00214833	8.999120E-11	ukb-b-19953	Body mass index (BMI)
rs56094641	0.07349670	0.03159390	ieu-b-18	0.0164458	115803	0.0547205	Multiple Sclerosis	461460	0.00201412	1.000000E-200	ukb-b-19953	Body mass index (BMI)
rs56133507	0.01376220	0.02819370	ieu-b-18	0.031384	115803	0.3690000	Multiple Sclerosis	461460	0.00247385	2.699980E-08	ukb-b-19953	Body mass index (BMI)
rs56143236	0.01266670	0.01369330	ieu-b-18	0.0186997	115803	0.4640000	Multiple Sclerosis	461460	0.00226533	2.300010E-08	ukb-b-19953	Body mass index (BMI)
rs56161855	0.02245750	0.02347340	ieu-b-18	0.0233181	115803	0.3141000	Multiple Sclerosis	461460	0.00291784	1.399910E-14	ukb-b-19953	Body mass index (BMI)
rs56203622	0.01797690	0.01521520	ieu-b-18	0.0235822	115803	0.5188000	Multiple Sclerosis	461460	0.00280183	1.400010E-10	ukb-b-19953	Body mass index (BMI)
rs56352336	-0.01632700	0.04698680	ieu-b-18	0.0243804	115803	0.0539498	Multiple Sclerosis	461460	0.00275021	2.900010E-09	ukb-b-19953	Body mass index (BMI)
rs56399737	-0.01613250	-0.01498710	ieu-b-18	0.0165271	115803	0.3645000	Multiple Sclerosis	461460	0.00199646	6.400300E-16	ukb-b-19953	Body mass index (BMI)
rs56858768	0.01591880	0.03045920	ieu-b-18	0.0180144	115803	0.0908699	Multiple Sclerosis	461460	0.00217378	2.399940E-13	ukb-b-19953	Body mass index (BMI)
rs56893062	0.01251410	-0.04296370	ieu-b-18	0.017679	115803	0.0150900	Multiple Sclerosis	461460	0.00215383	6.199980E-09	ukb-b-19953	Body mass index (BMI)
rs56930105	0.01575570	-0.00995033	ieu-b-18	0.0241322	115803	0.6801000	Multiple Sclerosis	461460	0.00286187	3.699990E-08	ukb-b-19953	Body mass index (BMI)
rs57636386	-0.04125530	-0.01557800	ieu-b-18	0.0303115	115803	0.6073000	Multiple Sclerosis	461460	0.00358326	1.100020E-30	ukb-b-19953	Body mass index (BMI)
rs57989773	0.01334880	0.03221330	ieu-b-18	0.0193635	115803	0.0961900	Multiple Sclerosis	461460	0.00236315	1.600000E-08	ukb-b-19953	Body mass index (BMI)
rs58862095	-0.02298770	0.02398540	ieu-b-18	0.0188993	115803	0.2044000	Multiple Sclerosis	461460	0.00200786	2.399940E-30	ukb-b-19953	Body mass index (BMI)
rs59068084	0.01105520	0.03324660	ieu-b-18	0.0170901	115803	0.0517297	Multiple Sclerosis	461460	0.00201048	3.799970E-08	ukb-b-19953	Body mass index (BMI)
rs59086897	0.03338500	0.02153010	ieu-b-18	0.0165542	115803	0.1934000	Multiple Sclerosis	461460	0.00196986	1.999860E-64	ukb-b-19953	Body mass index (BMI)
rs59227842	0.02296750	0.02245010	ieu-b-18	0.0174129	115803	0.1973000	Multiple Sclerosis	461460	0.00215313	1.500030E-26	ukb-b-19953	Body mass index (BMI)
rs594024	-0.01467710	0.00140098	ieu-b-18	0.0175572	115803	0.9364000	Multiple Sclerosis	461460	0.00199081	1.699810E-13	ukb-b-19953	Body mass index (BMI)
rs6023655	-0.01473460	-0.00230265	ieu-b-18	0.0198175	115803	0.9075000	Multiple Sclerosis	461460	0.00234997	3.599980E-10	ukb-b-19953	Body mass index (BMI)
rs60764613	0.02099000	-0.00419120	ieu-b-18	0.0233088	115803	0.8573000	Multiple Sclerosis	461460	0.00282688	1.100020E-13	ukb-b-19953	Body mass index (BMI)
rs61740466	-0.01351530	-0.03517410	ieu-b-18	0.0190356	115803	0.0646294	Multiple Sclerosis	461460	0.00231885	5.600030E-09	ukb-b-19953	Body mass index (BMI)
rs61813324	0.02902590	0.00994933	ieu-b-18	0.0369275	115803	0.7876010	Multiple Sclerosis	461460	0.00292038	2.800270E-23	ukb-b-19953	Body mass index (BMI)
rs61828641	0.02245850	-0.02508280	ieu-b-18	0.0253427	115803	0.3223000	Multiple Sclerosis	461460	0.00315913	1.200050E-12	ukb-b-19953	Body mass index (BMI)
rs61871615	-0.02671590	-0.00796817	ieu-b-18	0.0498877	115803	0.8731000	Multiple Sclerosis	461460	0.00359269	1.000000E-13	ukb-b-19953	Body mass index (BMI)
rs61903695	0.01662350	-0.00637961	ieu-b-18	0.0192242	115803	0.7400000	Multiple Sclerosis	461460	0.00227115	2.499770E-13	ukb-b-19953	Body mass index (BMI)
rs62020775	-0.01656650	0.02634400	ieu-b-18	0.0242336	115803	0.2770000	Multiple Sclerosis	461460	0.0028635	7.199960E-09	ukb-b-19953	Body mass index (BMI)
rs62072006	0.01564920	0.00300451	ieu-b-18	0.0231868	115803	0.8969000	Multiple Sclerosis	461460	0.00282374	2.999990E-08	ukb-b-19953	Body mass index (BMI)
rs62107261	-0.09115590	-0.10930300	ieu-b-18	0.101408	115803	0.2811000	Multiple Sclerosis	461460	0.00460882	4.600450E-87	ukb-b-19953	Body mass index (BMI)
rs62176243	-0.01496990	0.01587330	ieu-b-18	0.0188564	115803	0.3999000	Multiple Sclerosis	461460	0.00228878	6.099580E-11	ukb-b-19953	Body mass index (BMI)
rs62190049	-0.01117980	-0.01783990	ieu-b-18	0.0176927	115803	0.3133000	Multiple Sclerosis	461460	0.00203614	4.000000E-08	ukb-b-19953	Body mass index (BMI)
rs62241847	-0.01242630	-0.00259663	ieu-b-18	0.0173014	115803	0.8807000	Multiple Sclerosis	461460	0.00212897	5.300050E-09	ukb-b-19953	Body mass index (BMI)
rs62246311	0.02075390	-0.05826890	ieu-b-18	0.0269662	115803	0.0307100	Multiple Sclerosis	461460	0.00325487	1.799990E-10	ukb-b-19953	Body mass index (BMI)
rs62379271	0.01172370	-0.02146790	ieu-b-18	0.0169156	115803	0.2044000	Multiple Sclerosis	461460	0.00200557	5.000000E-09	ukb-b-19953	Body mass index (BMI)
rs62407562	0.01443930	-0.01823280	ieu-b-18	0.0193236	115803	0.3454000	Multiple Sclerosis	461460	0.00222463	8.499630E-11	ukb-b-19953	Body mass index (BMI)
rs6265	-0.03991850	-0.00722605	ieu-b-18	0.0206114	115803	0.7259000	Multiple Sclerosis	461460	0.00252719	3.299890E-56	ukb-b-19953	Body mass index (BMI)
rs6430068	0.01860660	0.01116210	ieu-b-18	0.0256305	115803	0.6632010	Multiple Sclerosis	461460	0.0031882	5.300050E-09	ukb-b-19953	Body mass index (BMI)
rs6444950	0.01585250	0.01024730	ieu-b-18	0.0189258	115803	0.5882000	Multiple Sclerosis	461460	0.00232007	8.300420E-12	ukb-b-19953	Body mass index (BMI)
rs6545714	-0.02052190	-0.01685710	ieu-b-18	0.0167007	115803	0.3128000	Multiple Sclerosis	461460	0.00201573	2.399940E-24	ukb-b-19953	Body mass index (BMI)
rs6560906	-0.01219800	-0.01948870	ieu-b-18	0.0178158	115803	0.2740000	Multiple Sclerosis	461460	0.00214136	1.200000E-08	ukb-b-19953	Body mass index (BMI)
rs6561937	-0.01593400	0.00757127	ieu-b-18	0.0198719	115803	0.7032000	Multiple Sclerosis	461460	0.00230346	4.600450E-12	ukb-b-19953	Body mass index (BMI)
rs6567160	0.05417230	0.00200200	ieu-b-18	0.0189133	115803	0.9157000	Multiple Sclerosis	461460	0.00234213	2.301440E-118	ukb-b-19953	Body mass index (BMI)
rs6575340	0.02073330	0.01734860	ieu-b-18	0.0169548	115803	0.3062000	Multiple Sclerosis	461460	0.00206189	8.699610E-24	ukb-b-19953	Body mass index (BMI)
rs6597975	0.01368100	-0.00380724	ieu-b-18	0.018552	115803	0.8374000	Multiple Sclerosis	461460	0.00199532	7.100680E-12	ukb-b-19953	Body mass index (BMI)
rs66679256	0.01488640	-0.02460010	ieu-b-18	0.016547	115803	0.1371000	Multiple Sclerosis	461460	0.00198759	6.899220E-14	ukb-b-19953	Body mass index (BMI)
rs6669341	-0.01702870	-0.02265470	ieu-b-18	0.0165755	115803	0.1717000	Multiple Sclerosis	461460	0.0019986	1.599930E-17	ukb-b-19953	Body mass index (BMI)
rs6682438	0.01315860	-0.05879500	ieu-b-18	0.0170461	115803	0.0005623	Multiple Sclerosis	461460	0.00210047	3.699990E-10	ukb-b-19953	Body mass index (BMI)
rs6705567	-0.01461680	-0.03025300	ieu-b-18	0.0169981	115803	0.0751104	Multiple Sclerosis	461460	0.00204919	9.799410E-13	ukb-b-19953	Body mass index (BMI)
rs6707827	0.01194370	0.01921420	ieu-b-18	0.0180462	115803	0.2870000	Multiple Sclerosis	461460	0.00217332	3.899960E-08	ukb-b-19953	Body mass index (BMI)
rs6710091	-0.01168350	0.00329457	ieu-b-18	0.0170074	115803	0.8464000	Multiple Sclerosis	461460	0.00206707	1.600000E-08	ukb-b-19953	Body mass index (BMI)
rs6713781	-0.01357360	-0.01891990	ieu-b-18	0.0169312	115803	0.2638000	Multiple Sclerosis	461460	0.00202799	2.199890E-11	ukb-b-19953	Body mass index (BMI)
rs6725931	0.01910460	-0.01257880	ieu-b-18	0.0232194	115803	0.5880000	Multiple Sclerosis	461460	0.00274403	3.299890E-12	ukb-b-19953	Body mass index (BMI)
rs6744646	0.05546840	0.00677698	ieu-b-18	0.0215087	115803	0.7527000	Multiple Sclerosis	461460	0.00261213	4.499870E-100	ukb-b-19953	Body mass index (BMI)
rs6752979	0.01252350	0.00461061	ieu-b-18	0.0177275	115803	0.7948000	Multiple Sclerosis	461460	0.00211721	3.299970E-09	ukb-b-19953	Body mass index (BMI)
rs67609008	0.01708630	0.02146790	ieu-b-18	0.0199587	115803	0.2821000	Multiple Sclerosis	461460	0.0022021	8.600030E-15	ukb-b-19953	Body mass index (BMI)
rs6769617	-0.01357540	0.02771240	ieu-b-18	0.0170507	115803	0.1041000	Multiple Sclerosis	461460	0.00208902	8.100280E-11	ukb-b-19953	Body mass index (BMI)
rs6774894	0.01330880	-0.02726840	ieu-b-18	0.017038	115803	0.1095000	Multiple Sclerosis	461460	0.00205674	9.700630E-11	ukb-b-19953	Body mass index (BMI)
rs6777784	0.01161250	0.00399202	ieu-b-18	0.017309	115803	0.8176000	Multiple Sclerosis	461460	0.00202753	1.000000E-08	ukb-b-19953	Body mass index (BMI)
rs6831088	-0.01152220	0.02537530	ieu-b-18	0.0170337	115803	0.1363000	Multiple Sclerosis	461460	0.0020591	2.199990E-08	ukb-b-19953	Body mass index (BMI)
rs6843852	0.01308970	-0.00320513	ieu-b-18	0.0164051	115803	0.8451000	Multiple Sclerosis	461460	0.00197607	3.500260E-11	ukb-b-19953	Body mass index (BMI)
rs6909685	-0.01461200	-0.03149070	ieu-b-18	0.0176314	115803	0.0740901	Multiple Sclerosis	461460	0.00211352	4.700020E-12	ukb-b-19953	Body mass index (BMI)
rs6922607	0.01488480	-0.04678820	ieu-b-18	0.0207305	115803	0.0240099	Multiple Sclerosis	461460	0.00251479	3.200000E-09	ukb-b-19953	Body mass index (BMI)
rs6938973	0.01822230	0.02665200	ieu-b-18	0.0166294	115803	0.1090000	Multiple Sclerosis	461460	0.00201872	1.800110E-19	ukb-b-19953	Body mass index (BMI)
rs6950388	0.01552190	0.00448991	ieu-b-18	0.033069	115803	0.8920000	Multiple Sclerosis	461460	0.00244806	2.300010E-10	ukb-b-19953	Body mass index (BMI)
rs6962980	-0.01595680	-0.01146550	ieu-b-18	0.0163633	115803	0.4834990	Multiple Sclerosis	461460	0.00198814	1.000000E-15	ukb-b-19953	Body mass index (BMI)
rs698147	-0.01285120	0.00059982	ieu-b-18	0.0169676	115803	0.9718000	Multiple Sclerosis	461460	0.00198514	9.600640E-11	ukb-b-19953	Body mass index (BMI)
rs7024334	-0.01381770	-0.02078250	ieu-b-18	0.0193577	115803	0.2830000	Multiple Sclerosis	461460	0.00238338	6.699930E-09	ukb-b-19953	Body mass index (BMI)
rs7027304	0.01454920	-0.00399202	ieu-b-18	0.0177444	115803	0.8220000	Multiple Sclerosis	461460	0.00208703	3.100270E-12	ukb-b-19953	Body mass index (BMI)
rs7034554	-0.01298260	0.02654930	ieu-b-18	0.0168819	115803	0.1158000	Multiple Sclerosis	461460	0.00204252	2.100000E-10	ukb-b-19953	Body mass index (BMI)
rs7038943	-0.01402020	-0.01084100	ieu-b-18	0.0169199	115803	0.5217000	Multiple Sclerosis	461460	0.00208641	1.800110E-11	ukb-b-19953	Body mass index (BMI)
rs704061	0.01463740	0.02439990	ieu-b-18	0.0162492	115803	0.1332000	Multiple Sclerosis	461460	0.00198552	1.699810E-13	ukb-b-19953	Body mass index (BMI)
rs7070670	-0.01232740	-0.01247750	ieu-b-18	0.0180836	115803	0.4902000	Multiple Sclerosis	461460	0.00211928	5.999980E-09	ukb-b-19953	Body mass index (BMI)
rs7081254	-0.01425160	0.01146550	ieu-b-18	0.0207791	115803	0.5811000	Multiple Sclerosis	461460	0.00245216	6.199980E-09	ukb-b-19953	Body mass index (BMI)
rs7124681	0.02569770	-0.02061100	ieu-b-18	0.0166734	115803	0.2164000	Multiple Sclerosis	461460	0.0020063	1.500030E-37	ukb-b-19953	Body mass index (BMI)
rs7132908	0.02979040	0.03998900	ieu-b-18	0.0169116	115803	0.0180501	Multiple Sclerosis	461460	0.00203363	1.399910E-48	ukb-b-19953	Body mass index (BMI)
rs71495038	0.02779980	0.01015140	ieu-b-18	0.0302039	115803	0.7368010	Multiple Sclerosis	461460	0.00370969	6.700390E-14	ukb-b-19953	Body mass index (BMI)
rs7201895	-0.01497810	-0.02439990	ieu-b-18	0.0177857	115803	0.1701000	Multiple Sclerosis	461460	0.00207999	6.000670E-13	ukb-b-19953	Body mass index (BMI)
rs7206608	0.01351220	0.03045920	ieu-b-18	0.0176747	115803	0.0848301	Multiple Sclerosis	461460	0.00211797	1.799990E-10	ukb-b-19953	Body mass index (BMI)
rs7218014	0.01895240	0.00856323	ieu-b-18	0.020101	115803	0.6701010	Multiple Sclerosis	461460	0.00249216	2.900010E-14	ukb-b-19953	Body mass index (BMI)
rs7232171	0.01234040	-0.01862550	ieu-b-18	0.016539	115803	0.2601000	Multiple Sclerosis	461460	0.00200821	8.000000E-10	ukb-b-19953	Body mass index (BMI)
rs723672	0.01112730	-0.03411150	ieu-b-18	0.0167468	115803	0.0416601	Multiple Sclerosis	461460	0.00200698	2.999990E-08	ukb-b-19953	Body mass index (BMI)
rs7250833	0.01350410	-0.01318660	ieu-b-18	0.0179433	115803	0.4624000	Multiple Sclerosis	461460	0.002188	6.699930E-10	ukb-b-19953	Body mass index (BMI)
rs7259070	0.02186900	-0.08929210	ieu-b-18	0.020604	115803	0.0000147	Multiple Sclerosis	461460	0.00203628	6.599330E-27	ukb-b-19953	Body mass index (BMI)
rs72634826	-0.02127700	-0.01775670	ieu-b-18	0.073032	115803	0.8079000	Multiple Sclerosis	461460	0.00228001	1.000000E-20	ukb-b-19953	Body mass index (BMI)
rs72649373	0.01780620	-0.04888540	ieu-b-18	0.0244483	115803	0.0455502	Multiple Sclerosis	461460	0.00287805	6.100000E-10	ukb-b-19953	Body mass index (BMI)
rs72673947	0.02188640	0.01298390	ieu-b-18	0.0263962	115803	0.6228010	Multiple Sclerosis	461460	0.00321496	9.899200E-12	ukb-b-19953	Body mass index (BMI)
rs72892910	0.03877980	-0.01212620	ieu-b-18	0.0216607	115803	0.5756000	Multiple Sclerosis	461460	0.00262053	1.500030E-49	ukb-b-19953	Body mass index (BMI)
rs72976986	-0.02322570	-0.03411150	ieu-b-18	0.0230557	115803	0.1390000	Multiple Sclerosis	461460	0.00254712	7.599760E-20	ukb-b-19953	Body mass index (BMI)
rs73026725	-0.02231800	-0.01783990	ieu-b-18	0.0226642	115803	0.4312000	Multiple Sclerosis	461460	0.00275052	4.900040E-16	ukb-b-19953	Body mass index (BMI)
rs73052033	-0.03039290	0.00631993	ieu-b-18	0.0213294	115803	0.7669990	Multiple Sclerosis	461460	0.00254634	7.700160E-33	ukb-b-19953	Body mass index (BMI)
rs7306534	-0.01127540	-0.02634400	ieu-b-18	0.0167835	115803	0.1165000	Multiple Sclerosis	461460	0.00205437	4.099960E-08	ukb-b-19953	Body mass index (BMI)
rs73124396	-0.01544720	-0.00309520	ieu-b-18	0.0200971	115803	0.8776000	Multiple Sclerosis	461460	0.0024548	3.099990E-10	ukb-b-19953	Body mass index (BMI)
rs73142879	-0.02669360	0.05911330	ieu-b-18	0.0212957	115803	0.0055061	Multiple Sclerosis	461460	0.00252257	3.599980E-26	ukb-b-19953	Body mass index (BMI)
rs73193736	-0.01771650	-0.01252130	ieu-b-18	0.0193421	115803	0.5173990	Multiple Sclerosis	461460	0.00232002	2.199890E-14	ukb-b-19953	Body mass index (BMI)
rs73213484	-0.02257660	-0.04840910	ieu-b-18	0.0231983	115803	0.0369097	Multiple Sclerosis	461460	0.0028364	1.699810E-15	ukb-b-19953	Body mass index (BMI)
rs7331420	-0.01437020	-0.00030005	ieu-b-18	0.0159591	115803	0.9850000	Multiple Sclerosis	461460	0.00220081	6.599330E-11	ukb-b-19953	Body mass index (BMI)
rs7357754	0.01411460	0.05675830	ieu-b-18	0.0165269	115803	0.0005941	Multiple Sclerosis	461460	0.00198362	1.100020E-12	ukb-b-19953	Body mass index (BMI)
rs73601548	0.01773540	0.01419870	ieu-b-18	0.0256783	115803	0.5803010	Multiple Sclerosis	461460	0.00311639	1.299990E-08	ukb-b-19953	Body mass index (BMI)
rs73985439	0.01366160	0.00501254	ieu-b-18	0.0179707	115803	0.7803010	Multiple Sclerosis	461460	0.00214059	1.700000E-10	ukb-b-19953	Body mass index (BMI)
rs7442885	-0.02281630	0.00350614	ieu-b-18	0.0197365	115803	0.8590000	Multiple Sclerosis	461460	0.00241251	3.199630E-21	ukb-b-19953	Body mass index (BMI)
rs745249	0.01761180	0.00149888	ieu-b-18	0.0182659	115803	0.9346000	Multiple Sclerosis	461460	0.00219724	1.100020E-15	ukb-b-19953	Body mass index (BMI)
rs7498665	0.02686460	0.01836770	ieu-b-18	0.0172726	115803	0.2876000	Multiple Sclerosis	461460	0.00201997	2.299850E-40	ukb-b-19953	Body mass index (BMI)
rs7516554	0.01200660	0.00110061	ieu-b-18	0.0166196	115803	0.9472000	Multiple Sclerosis	461460	0.00201565	2.599980E-09	ukb-b-19953	Body mass index (BMI)
rs7519259	0.01400280	-0.00786896	ieu-b-18	0.0161179	115803	0.6254000	Multiple Sclerosis	461460	0.00198381	1.699810E-12	ukb-b-19953	Body mass index (BMI)
rs754635	0.02199250	0.01093990	ieu-b-18	0.026541	115803	0.6802010	Multiple Sclerosis	461460	0.0031093	1.500030E-12	ukb-b-19953	Body mass index (BMI)
rs75499503	-0.01803310	-0.03072320	ieu-b-18	0.0193047	115803	0.1115000	Multiple Sclerosis	461460	0.00241845	8.900200E-14	ukb-b-19953	Body mass index (BMI)
rs7568228	-0.01196250	0.01705380	ieu-b-18	0.0168224	115803	0.3107000	Multiple Sclerosis	461460	0.00197288	1.299990E-09	ukb-b-19953	Body mass index (BMI)
rs7571496	-0.01589160	-0.02829590	ieu-b-18	0.0181895	115803	0.1198000	Multiple Sclerosis	461460	0.00225454	1.800110E-12	ukb-b-19953	Body mass index (BMI)
rs76183894	-0.02195840	0.03573080	ieu-b-18	0.0449625	115803	0.4268000	Multiple Sclerosis	461460	0.00364437	1.700000E-09	ukb-b-19953	Body mass index (BMI)
rs7619139	0.01345370	0.00786896	ieu-b-18	0.0170694	115803	0.6448000	Multiple Sclerosis	461460	0.00201061	2.199890E-11	ukb-b-19953	Body mass index (BMI)
rs765874	-0.01205920	0.01143440	ieu-b-18	0.0163077	115803	0.4832000	Multiple Sclerosis	461460	0.00197569	1.000000E-09	ukb-b-19953	Body mass index (BMI)
rs76702514	-0.01648620	0.01308520	ieu-b-18	0.0205903	115803	0.5251000	Multiple Sclerosis	461460	0.00243278	1.200050E-11	ukb-b-19953	Body mass index (BMI)
rs7683836	-0.01224690	-0.01653600	ieu-b-18	0.0164538	115803	0.3149000	Multiple Sclerosis	461460	0.00199427	8.199930E-10	ukb-b-19953	Body mass index (BMI)
rs7704382	0.01201260	-0.00339423	ieu-b-18	0.0163356	115803	0.8354000	Multiple Sclerosis	461460	0.00199631	1.799990E-09	ukb-b-19953	Body mass index (BMI)
rs7708584	-0.01593210	-0.00628024	ieu-b-18	0.0164543	115803	0.7027000	Multiple Sclerosis	461460	0.0019949	1.399910E-15	ukb-b-19953	Body mass index (BMI)
rs7761673	-0.01359150	-0.00149888	ieu-b-18	0.0195863	115803	0.9390000	Multiple Sclerosis	461460	0.0023901	1.299990E-08	ukb-b-19953	Body mass index (BMI)
rs7762794	0.01490770	-0.00803217	ieu-b-18	0.017723	115803	0.6504010	Multiple Sclerosis	461460	0.00218606	9.099130E-12	ukb-b-19953	Body mass index (BMI)
rs7774	0.01498800	-0.02664190	ieu-b-18	0.0185664	115803	0.1513000	Multiple Sclerosis	461460	0.00215169	3.299890E-12	ukb-b-19953	Body mass index (BMI)
rs7776021	0.01237010	-0.01508560	ieu-b-18	0.0182828	115803	0.4093000	Multiple Sclerosis	461460	0.00218251	1.400010E-08	ukb-b-19953	Body mass index (BMI)
rs7802342	0.01227480	0.01146550	ieu-b-18	0.0183282	115803	0.5316000	Multiple Sclerosis	461460	0.00218145	1.799990E-08	ukb-b-19953	Body mass index (BMI)
rs7805441	0.01337470	-0.00498754	ieu-b-18	0.0161189	115803	0.7570000	Multiple Sclerosis	461460	0.00198884	1.800110E-11	ukb-b-19953	Body mass index (BMI)
rs784257	0.01793150	-0.02552300	ieu-b-18	0.0211034	115803	0.2265000	Multiple Sclerosis	461460	0.00254968	1.999860E-12	ukb-b-19953	Body mass index (BMI)
rs78605811	-0.03273700	-0.03546370	ieu-b-18	0.0394189	115803	0.3683000	Multiple Sclerosis	461460	0.00444673	1.800110E-13	ukb-b-19953	Body mass index (BMI)
rs7893571	0.01405530	0.02932580	ieu-b-18	0.0173376	115803	0.0907507	Multiple Sclerosis	461460	0.00210152	2.299850E-11	ukb-b-19953	Body mass index (BMI)
rs7924036	-0.01428360	-0.00290421	ieu-b-18	0.0163248	115803	0.8588000	Multiple Sclerosis	461460	0.0019778	5.100350E-13	ukb-b-19953	Body mass index (BMI)
rs7925100	0.01472500	-0.03874080	ieu-b-18	0.016884	115803	0.0217601	Multiple Sclerosis	461460	0.0020221	3.299890E-13	ukb-b-19953	Body mass index (BMI)
rs7944782	0.01576060	0.00269636	ieu-b-18	0.0164245	115803	0.8696000	Multiple Sclerosis	461460	0.00198748	2.199890E-15	ukb-b-19953	Body mass index (BMI)
rs7947143	-0.01827820	0.00250313	ieu-b-18	0.0223687	115803	0.9109000	Multiple Sclerosis	461460	0.00267522	8.300420E-12	ukb-b-19953	Body mass index (BMI)
rs7996639	0.01455590	-0.01592620	ieu-b-18	0.0163325	115803	0.3295000	Multiple Sclerosis	461460	0.00200245	3.599980E-13	ukb-b-19953	Body mass index (BMI)
rs80135274	0.02133900	0.00200200	ieu-b-18	0.0333977	115803	0.9522000	Multiple Sclerosis	461460	0.00388113	3.799970E-08	ukb-b-19953	Body mass index (BMI)
rs8015400	0.02134220	0.00498754	ieu-b-18	0.0172907	115803	0.7729990	Multiple Sclerosis	461460	0.00211709	6.700390E-24	ukb-b-19953	Body mass index (BMI)
rs8020365	0.02510680	0.02716570	ieu-b-18	0.0198249	115803	0.1706000	Multiple Sclerosis	461460	0.00239517	1.000000E-25	ukb-b-19953	Body mass index (BMI)
rs8024137	0.01563290	0.05078820	ieu-b-18	0.0237554	115803	0.0325200	Multiple Sclerosis	461460	0.00276778	1.600000E-08	ukb-b-19953	Body mass index (BMI)
rs8025516	-0.01465580	0.01173090	ieu-b-18	0.0169118	115803	0.4879000	Multiple Sclerosis	461460	0.00207578	1.699810E-12	ukb-b-19953	Body mass index (BMI)
rs8076669	0.01406810	-0.00339423	ieu-b-18	0.0162555	115803	0.8346000	Multiple Sclerosis	461460	0.00199601	1.800110E-12	ukb-b-19953	Body mass index (BMI)
rs8089514	0.01298800	0.00461061	ieu-b-18	0.0169005	115803	0.7850010	Multiple Sclerosis	461460	0.00207588	3.899960E-10	ukb-b-19953	Body mass index (BMI)
rs8112818	-0.02069600	-0.03252320	ieu-b-18	0.0175766	115803	0.0642599	Multiple Sclerosis	461460	0.00202712	1.800110E-24	ukb-b-19953	Body mass index (BMI)
rs8132491	-0.01537500	0.01663760	ieu-b-18	0.0202155	115803	0.4105000	Multiple Sclerosis	461460	0.00219317	2.399940E-12	ukb-b-19953	Body mass index (BMI)
rs815163	-0.01648500	-0.01872360	ieu-b-18	0.0163177	115803	0.2512000	Multiple Sclerosis	461460	0.00198582	1.000000E-16	ukb-b-19953	Body mass index (BMI)
rs852042	-0.01312070	0.00598207	ieu-b-18	0.0194155	115803	0.7580000	Multiple Sclerosis	461460	0.00231309	1.400010E-08	ukb-b-19953	Body mass index (BMI)
rs862320	-0.02317030	-0.00079968	ieu-b-18	0.0156702	115803	0.9593000	Multiple Sclerosis	461460	0.00201345	1.200050E-30	ukb-b-19953	Body mass index (BMI)
rs879620	0.02411360	0.00558438	ieu-b-18	0.01697	115803	0.7420990	Multiple Sclerosis	461460	0.00203595	2.299850E-32	ukb-b-19953	Body mass index (BMI)
rs909892	-0.01841050	-0.03816250	ieu-b-18	0.0247834	115803	0.1236000	Multiple Sclerosis	461460	0.00291255	2.599980E-10	ukb-b-19953	Body mass index (BMI)
rs923994	-0.01457610	0.00558438	ieu-b-18	0.0195724	115803	0.7754000	Multiple Sclerosis	461460	0.00240219	1.299990E-09	ukb-b-19953	Body mass index (BMI)
rs9267671	0.02614900	-0.08470870	ieu-b-18	0.0393122	115803	0.0311803	Multiple Sclerosis	461460	0.00413587	2.599980E-10	ukb-b-19953	Body mass index (BMI)
rs9291822	-0.01426300	-0.02716570	ieu-b-18	0.0163396	115803	0.0964007	Multiple Sclerosis	461460	0.00199464	8.600030E-13	ukb-b-19953	Body mass index (BMI)
rs9294260	0.01478200	0.03252320	ieu-b-18	0.0164263	115803	0.0477101	Multiple Sclerosis	461460	0.00198818	1.000000E-13	ukb-b-19953	Body mass index (BMI)
rs9349235	0.01118090	-0.01192860	ieu-b-18	0.0164144	115803	0.4674000	Multiple Sclerosis	461460	0.00200924	2.599980E-08	ukb-b-19953	Body mass index (BMI)
rs935166	-0.01610660	-0.00915794	ieu-b-18	0.0165054	115803	0.5790010	Multiple Sclerosis	461460	0.00197292	3.199630E-16	ukb-b-19953	Body mass index (BMI)
rs9388446	0.01110130	-0.01480910	ieu-b-18	0.0163035	115803	0.3637000	Multiple Sclerosis	461460	0.00197919	2.000000E-08	ukb-b-19953	Body mass index (BMI)
rs9463175	-0.01150000	-0.01734860	ieu-b-18	0.0170815	115803	0.3098000	Multiple Sclerosis	461460	0.00210115	4.399970E-08	ukb-b-19953	Body mass index (BMI)
rs9478496	0.01818360	0.00280393	ieu-b-18	0.0219601	115803	0.8984000	Multiple Sclerosis	461460	0.00267493	1.100020E-11	ukb-b-19953	Body mass index (BMI)
rs9515446	0.01510510	0.01765490	ieu-b-18	0.0168904	115803	0.2959000	Multiple Sclerosis	461460	0.00199052	3.199630E-14	ukb-b-19953	Body mass index (BMI)
rs9522180	-0.01410950	-0.00189820	ieu-b-18	0.016982	115803	0.9110000	Multiple Sclerosis	461460	0.00199325	1.500030E-12	ukb-b-19953	Body mass index (BMI)
rs9571687	-0.01353880	-0.00080032	ieu-b-18	0.0176827	115803	0.9639000	Multiple Sclerosis	461460	0.0021094	1.400010E-10	ukb-b-19953	Body mass index (BMI)
rs9638713	-0.03617350	-0.10602700	ieu-b-18	0.0573543	115803	0.0645104	Multiple Sclerosis	461460	0.00635399	1.200000E-08	ukb-b-19953	Body mass index (BMI)
rs9830592	0.01548490	0.01950850	ieu-b-18	0.0164939	115803	0.2369000	Multiple Sclerosis	461460	0.00200164	1.000000E-14	ukb-b-19953	Body mass index (BMI)
rs9839081	-0.01170100	0.00722605	ieu-b-18	0.0188193	115803	0.7010000	Multiple Sclerosis	461460	0.00214045	4.600020E-08	ukb-b-19953	Body mass index (BMI)
rs9843653	0.02945090	0.01045450	ieu-b-18	0.0163755	115803	0.5232000	Multiple Sclerosis	461460	0.00197538	2.900010E-50	ukb-b-19953	Body mass index (BMI)
rs9876664	-0.01804780	-0.00528600	ieu-b-18	0.0169466	115803	0.7550990	Multiple Sclerosis	461460	0.00204174	9.600640E-19	ukb-b-19953	Body mass index (BMI)
rs9888533	0.01200380	0.01521520	ieu-b-18	0.0209793	115803	0.4683000	Multiple Sclerosis	461460	0.0020182	2.699980E-09	ukb-b-19953	Body mass index (BMI)
rs9926784	-0.02381800	-0.03748840	ieu-b-18	0.0214731	115803	0.0808407	Multiple Sclerosis	461460	0.00254677	8.600030E-21	ukb-b-19953	Body mass index (BMI)
rs9951619	0.01442310	-0.01806210	ieu-b-18	0.0188867	115803	0.3389000	Multiple Sclerosis	461460	0.00235777	9.499920E-10	ukb-b-19953	Body mass index (BMI)

Supplementary File 2
